# Design, synthesis, and *in vitro*, *in vivo*, and *in silico* evaluation of novel substituted 1,3,4-thiadiazole derivatives as anticonvulsant agents

**DOI:** 10.3389/fchem.2024.1515866

**Published:** 2025-02-12

**Authors:** Tulika Anthwal, Swati Pant, Preeti Rana, Sumitra Nain

**Affiliations:** ^1^ Department of Pharmacy, Banasthali Vidyapith, Banasthali, Rajasthan, India; ^2^ National Institute of Pharmaceutical Education and Research (NIPER), Hyderabad, Telangana, India

**Keywords:** 1,3,4-thiadiazoles, carbonic anhydrase, anticonvulsant activity, maximal electroshock seizure, subcutaneous pentylenetetrazole

## Abstract

In the present study, a library of ten novel substituted 1,3,4‐thiadiazole derivatives were designed and synthesized using an appropriate synthetic route. The characterization of the synthesized compounds was performed by FT-IR and NMR (^1^H and ^13^C) spectroscopy. The synthesized compounds were assayed for *in vitro* human carbonic anhydrase (CA) inhibition against two isoforms II and IX. The neurotoxicity of the synthesized derivatives was also evaluated using the rotarod test, along with their *in vivo* anticonvulsant activity, which was determined using the maximal electroshock seizure (MES) and subcutaneous pentylenetetrazole (sc-PTZ) methods. Although all the compounds showed good CA inhibition and anticonvulsant activity, two compounds **6d** and **7d** showed the highest CA inhibition and anticonvulsant activity in both the isoforms and tested methods compared to the standard drugs (sodium valproate and acetazolamide), without any sign of neurotoxicity observed at the highest dose (300 mg/kg). Similarly, the standard drugs also displayed no neurotoxicity at the highest dose (300 mg/kg). Furthermore, the potent compounds (**6d** and **7d**) were evaluated for the biochemical parameters, such as lipid peroxidation, nitrite oxide, reduced glutathione, superoxide dismutase, and total antioxidant capacity, and the GABA level was also determined. Finally, compound **6d** was docked against CA-II and CA-IX (PDB-ID-5SZ5 and 5AML) receptors. The study concluded that the compounds **6d** and **7d** can be considered potent anticonvulsant agents for future research.

## Graphical Abstract

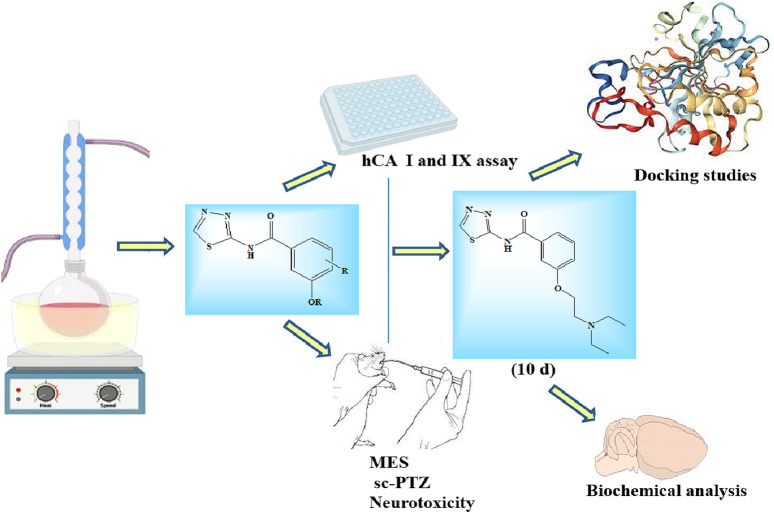

## Highlights

• Ten novel 1,3,4-thiadiazole derivatives were synthesized, characterized, and evaluated for *in vitro* and *in vivo* anticonvulsant activity and found to have potent anticonvulsant action by inhibiting human carbonic anhydrase (CA) with no neurotoxicity observed.
• Two compounds **6d** and **7d** displayed excellent anticonvulsant activity in maximal electroshock seizure (MES) and subcutaneous pentylenetetrazole (sc-PTZ) seizure models with long-duration action at minimum dose and were further evaluated for several biochemical parameters.
• Compound **6d** was further docked against CA-II and CA-IX enzymes.

## 1 Introduction

Epilepsy is a neurological condition that affects over 60 million people worldwide (Ngugi et al., 2010; Beghi and Giussani, 2018). It is characterized by recurrent seizures that arise from an uncontrollably high voltage in a group of brain cells (Goldenberg, 2010; Antuono et al., 2010). As per the World Health Organization (WHO), approximately 80 out of every 100 people who have epilepsy reside in undeveloped nations, the majority of whom lack access to quality healthcare (Epilepsy, 2024, WHO). The search for an effective treatment for epilepsy has spurred notable breakthroughs in suitable medication with a minimal chance of adverse effects.

Anticonvulsant medications that are approved as antiepileptic medicine just provide symptomatic treatment. Furthermore, the majority of these current drugs have a suboptimal efficacy rate (less than 70%) and are associated with a range of negative side effects (Song and Deng, 2018). However, since medication is considered the sole effective treatment for epilepsy, many people do not show improvement with monotherapy and require polytherapy involving many, often less effective, anticonvulsants. These limitations precisely explain the need for further investigations to find new and effective anticonvulsant agents.

A wide range of medicinal substances with varying molecular structures that are approved for use in clinical purpose as anticonvulsants include hydantoins, barbiturates, benzodiazepines (BZDs), succinimide, valproate, imides, oxazolidinediones, sulfonamides, and gamma-amino analogs of aminobutyric acid (GABA) (Aboul‐Enein et al., 2012a). Benzodiazepines are the significant class of anticonvulsants with strong anti-generalized epilepsy effects, achieved by binding to GABA_A_ ion channels. This binding increases the chloride ion’s frequency channel opening, which makes GABA’s inhibitory actions easier to achieve, but these medications have several negative side effects, including sedation, memory loss, and tolerance development (Rudolph and Knoflach, 2011). The discovery of novel anticonvulsant drugs with minimal adverse effect on memory has been an important area in drug developmentover the last 10 years (Ayati et al., 2016).

Numerous five- and six-membered heterocyclic compounds have been documented to exhibit anticonvulsant properties, including triazoles, pyrrolidines, oxadiazoles, and thiadiazoles (Kharb et al., 2011; Riley and Jackson, 2016; Kasireddy Sudarshan et al., 2019). In particular, thiadiazole derivatives have been extensively utilized as anticonvulsant medications. Several pharmaceuticals and commercial medications contain thiadiazole, a versatile five-membered heterocyclic molecule having four isomeric structures: 1,2,3-thiadiazole; 1,2,4-thiadiazole; 1,2,5-thiadiazole; and 1,3,4-thiadiazole (Dawood and Farghaly, 2017; Kushwaha et al., 2012).

Among these isomers, 1,3,4-thiadiazoles play a well-established role as pharmacologically significant scaffolds, exhibiting a wide range of activities, including enzyme inhibition and human platelet aggregation inhibition, and antimicrobial, anti-inflammatory, antitumor, antioxidant, and antiviral properties. Furthermore, numerous compounds containing 1,3,4-thiadiazole have been produced and assessed for their potential as anticonvulsant drugs (Shawali, 2014; Haider et al., 2015; Hu et al., 2014; Serban et al., 2018; Almasirad et al., 2007).

## 2 Materials and methods

### 2.1 Chemistry

#### 2.1.1 General

All raw ingredients and solvents utilized in this study were of AR grade, obtained from E. Merck Limited, Sigma-Aldrich, and Tokyo Chemical Industry (TCI), and they were used directly for synthesis without any purification. All reactions were principally monitored using TLCs (60 F_254_, Merck, 0.25-mm thickness) with ethyl acetate: hexane (1:1) as the solvent system, and the spots were observed using UV light (Per fit, India) or staining methods such as iodine and ninhydrin. Further column chromatography was performed to purify the synthesized compounds, and the yields stated are based on isolated pure products rather than confirmed reaction conditions. The melting points were determined in open glass capillary tubes using a melting point apparatus (Veego, model: VMP-D) and were uncorrected. The FT-IR spectra were recorded in KBr pellets on the FT-IR spectrophotometer (Bruker Tech. Pvt. Ltd). The NMR analysis was conducted using the Bruker Advance-II D800 (400 MHz) Spectrometer in deuterated solvent DMSO-*d*_*6*_ at 400 MHz. All chemical shifts (δ) are expressed in parts per million (ppm) relative to the standard TMS, and the peaks are designated as singlets (s), doublets (d), double doublets (dd), triplets (t), and multiplets (m).

#### 2.1.2 General procedure for the synthesis of intermediate compounds 4 and 5

The synthesis of the new derivatives of 1,3,4-thiadiazole was performed according to the earlier literature with slight modifications (Yang et al., 2012). In brief, substituted benzoic acid (1.15 mM) was dissolved in 10 mL of DMF at 0°C. After completely dissolving it, EDCI (1.25 mM) and HOBt (1.25 mM) were added, and the reaction mixture was stirred for 30 min at room temperature. After 30 min, an ice-cooled solution of 2-amino-1,3,4-thiadiazole (1.25 mM) in 10 mL of DMF was added dropwise to the reaction mixture, and the reaction was stirred at room temperature for 6 hours (the reaction was carried out under a nitrogen environment). The completion of the reaction was monitored via TLC taking ethyl acetate: hexane (1:1) as a solvent. After confirmation, the final compounds were extracted with ethyl acetate, water, and saturated NaHCO_3_ and washed several times. Subsequently, the organic layer was separated, and the excess solvent was removed under vacuum, dried, and recrystallized using ethanol.

#### 2.1.3 General procedure for the synthesis of final compounds 6 (a–e) and 7 (a–e)

The synthesis of the new derivatives of 1,3,4-thiadiazole was performed according to the earlier literature (Mondal and Mallik, 2014) with slight modifications. In brief, equimolar quantities of intermediate (**4, 5**) and various substituted amines were dissolved in DMF (6 mL) in the presence of anhydrous potassium carbonate (0.08 g) and were refluxed at 140°C for 4 hours (the reaction was carried out under a nitrogen environment). The completion of the reaction was monitored via TLC taking ethyl acetate: hexane (1:1) as a solvent. After confirmation, the final compounds were extracted with ethyl acetate, water, and saturated NaHCO_3_. Subsequently, the organic layer was separated, the excess solvent was removed under vacuum, and the solid precipitate was obtained, scrapped off, and recrystallized with ethanol. Further purification of the compound was performed via column chromatography.

##### 2.1.3.1 3-Hydroxy-N-(1,3,4-thiadiazol-2-yl)benzamide (4)

Compound: C_9_H_7_N_3_O_2_S; molecular weight: 221.24; cream crystalline solid; yield: 72%; and melting point: 154°C–156°C. The FT-IR spectrum (KBr, ν_max_, cm^-1^) shows significant peaks at 3430 (N-H stretching), 3311 (O-H bending, aromatic), 2930 (C-H stretching, aromatic), 1784 (C=O stretching, aromatic), 1620 (C-C stretching, aromatic), 1514 (C=C stretching, aromatic), 749(C-S stretching). The 1H NMR spectrum data (400 MHz, DMSO-d_6_, δ, ppm) displays the following signals: 8.58 (s, 1H), 7.95 (d, *J* = 8.4 Hz, 1H), 7.67 (d, *J* = 8.3 Hz, 1H), 7.55–7.47 (m, 1H), 7.39 (ddd, *J* = 8.1, 6.8, and 1.1 Hz, 1H), 7.22 (s, 1H), and 3.57 (s, 1H). The 13C NMR spectrum (101 MHz, DMSO-d_6_, δ, ppm) displays peaks at 168.76, 143.26, 143.15, 128.25, 127.44, 124.84, 119.47, 110.22, and 55.04.

##### 2.1.3.2 3-Hydroxy-4-methoxy-N-(1,3,4-thiadiazol-2-yl)benzamide (5)

Compound: C_10_H_9_N_3_O_3_S; molecular weight: 251.26; brown crystalline solid; yield: 76%; and melting point: 164°C–168°C. The FT-IR spectrum (KBr, ν_max_, cm^-1^) shows significant peaks at 3422 (N-H stretching), 3189 (O-H bending, aromatic), 2938 (C-H stretching, aromatic), 2866 (O-CH_3_ stretching), 1856 (C=N stretching), 1677 (C=O stretching, aromatic), 1540 (C-C stretching, aromatic), 1445 (C=C stretching, aromatic), and 743(C-S stretching). The ^1^H NMR spectrum (400 MHz, DMSO-d_6_, δ, ppm) displays the following signals: 9.20 (s, 1H), 8.58 (s, 1H), 7.90 (d, *J* = 8.4 Hz, 1H), 7.43 (d, *J* = 2.3 Hz, 1H), 7.38 (d, *J* = 2.1 Hz, 1H), 6.98 (d, *J* = 8.5 Hz, 1H), and 3.81 (s, 3H). The ^13^C NMR spectrum (101 MHz, DMSO-d_6_, δ, ppm) reveals peaks at 167.75, 152.03, 149.29, 146.54, 143.28, 128.21, 126.69, 111.72, 110.45, and 56.03.

##### 2.1.3.3 3-(2-morpholinoethoxy)-N-(1,3,4-thiadiazol-2-yl)benzamide (6a)

Compound: C_15_H_18_N_4_O_3_S; molecular weight: 334.39; light yellow crystalline solid; yield: 74%; and melting point: 172°C–176°C. The FT-IR spectrum (KBr, ν_max_, cm^-1^) shows significant peaks at 3412 (N-H stretching), 3275 (C-H stretching, aromatic), 2926 (C-H stretching), 1781 (C=N stretching), 1626 (C=O stretching), 1501 (C-C stretching), 1454 (C=C stretching), 1269 (C-O stretching), and 750 (C-S stretching). The ^1^H NMR spectrum (400 MHz, DMSO-d_6_, δ, ppm) displays the following signals: 9.13 (s, 1H), 7.98 (d, *J* = 8.5 Hz, 2H), 7.29–7.08 (m, 2H), 4.59 (s, 1H), 4.30 (t, *J* = 5.7 Hz, 2H), 4.06 (t, *J* = 5.7 Hz, 4H), 3.36 (t, *J* = 4.6 Hz, 2H), and 2.29 (d, *J* = 4.7 Hz, 4H). The ^13^C NMR spectrum (101 MHz, DMSO-d_6_, δ, ppm) reveals peaks at 167.34, 165.18, 158.12, 157.03, 142.42, 130.75, 127.88, 114.30, 65.62, 61.77, and 53.21.

##### 2.1.3.4 3-(2-(piperidin-1-yl)ethoxy)-N-(1,3,4-thiadiazol-2-yl)benzamide (6b)

Compound: C_16_H_20_N_4_O_2_S; molecular weight: 332.42; yellow crystalline solid; yield: 71%; and melting point: 225°C–228°C. The FT-IR spectrum (KBr, ν_max_, cm^-1^) shows significant peaks at 3428 (N-H stretching), 3266 (C-H stretching, aromatic), 2932 (C-H stretching, alkyl), 1758 (C=N stretching), 1634 (C=O stretching), 1527 (C-C stretching), 1474 (C=C stretching), 1257 (C-O stretching), and 749 (C-S stretching). The ^1^H NMR spectrum (400 MHz, DMSO-d_6_, δ, ppm) displays the following signals: 9.30 (s, 1H), 7.95 (d, *J* = 8.4 Hz, 1H), 7.55 (d, *J* = 2.7 Hz, 1H), 7.55–7.09 (m, 1H), 7.08 (ddd, *J* = 8.1, 2.5, 1.2 Hz, 1H), 4.23 (t, *J* = 5.7 Hz, 2H), 3.66 (s, 1H), 3.53 (t, *J* = 4.6 Hz, 2H), 2.79 (dd, *J* = 6.3, 3.7 Hz, 4H), 1.30 (d, *J* = 3.8 Hz, 4H), and 1.08 (t, *J* = 7.1 Hz, 2H). The ^13^C NMR spectrum (101 MHz, DMSO-d_6_, δ, ppm) reveals peaks at 165.98, 158.92, 157.83, 143.22, 131.55, 130.45, 128.68, 127.85, 115.10, 110.04, 62.57, 54.01, 53.55, and 16.07.

##### 2.1.3.5 3-(2-(pyrrolidin-1-yl)ethoxy)-N-(1,3,4-thiadiazol-2-yl)benzamide (6c)

Compound: C_15_H_18_N_4_O_2_S; molecular weight: 318.39; dark yellow crystalline solid; yield: 74%, and melting point: 215°C–218°C. The FT-IR spectrum (KBr, ν_max_, cm^-1^) shows significant peaks at 3428 (N-H stretching), 3288 (C-H stretching, aromatic), 2930 (C-H stretching, alkyl), 1789 (C=N stretching), 1617 (C=O stretching), 1537 (C-C stretching), 1403 (C=C stretching) 1270 (C-O stretching), and 741 (C-S stretching). The ^1^H NMR spectrum (400 MHz, DMSO-d_6_, δ, ppm) displays the following signals: 9.40 (s, 1H), 8.05 (d, *J* = 8.3 Hz, 1H), 7.73 (d, *J* = 7.9 Hz, 1H), 7.68–7.47 (m, 1H), 7.44 (s, 1H), 7.45–6.67 (m, 2H), 4.33 (s, 1H), 3.76 (t, *J* = 5.7 Hz, 2H), 3.63 (t, *J* = 7.3 Hz, 2H), 2.89 (d, *J* = 2.3 Hz, 2H), and 1.40 (d, *J* = 3.5 Hz, 4H). The ^13^C NMR spectrum (101 MHz, DMSO-d_6_, δ, ppm) reveals peaks at 166.01, 159.92, 157.83, 143.24, 130.45, 129.89, 125.35, 120.00, 115.60, 110.04, 66.42, 56.39, and 54.01.

##### 2.1.3.6 3-(2-(diethylamino)ethoxy)-N-(1,3,4-thiadiazol-2-yl)benzamide (6d)

Compound: C_15_H_20_N_4_O_2_S; molecular weight: 320.41; pale yellow crystalline solid; yield: 70%, and melting point: 205°C–208°C. The FT-IR spectrum (KBr, ν_max_, cm^-1^) shows significant peaks at 3412 (N-H stretching), 3284 (C-H stretching, aromatic), 2929 (C-H stretching, alkyl), 2858 (N-CH_2_-CH_3_ stretching), 1790 (C=N stretching), 1630 (C=O stretching), 1517 (C-C stretching), 1454 (C=C stretching), 1285(C-O stretching), and 745 (C-S str).The ^1^H NMR spectrum (400 MHz, DMSO-d_6_, δ, ppm) displays the following signals: 9.43 (s, 1H), 8.28 (d, *J* = 8.5 Hz, 1H), 7.85 (ddd, *J* = 8.2, 6.9, and 1.0 Hz, 1H), 7.74–7.62 (m, 1H), 7.47 (dd, *J* = 8.6 and 7.4 Hz, 1H), 5.11–4.80 (m, 2H), 4.36 (s, 1H), 3.66 (t, *J* = 4.6 Hz, 2H), 2.92 (dd, *J* = 6.2 and 4.0 Hz, 4H), and 1.21 (t, *J* = 7.2 Hz, 6H). The ^13^C NMR spectrum (101 MHz, DMSO-d_6_, δ, ppm) reveals peaks at 167.14, 164.98, 156.83, 142.22, 130.55, 129.45, 127.68, 114.10, 109.04, 61.57, 53.01, 52.55, and 15.07.

##### 2.1.3.7 3-(2-(dimethylamino)ethoxy)-N-(1,3,4-thiadiazol-2-yl)benzamide (6e)

Compound: C_13_H_16_N_4_O_2_S; molecular weight: 334.39; yellow crystalline solid; yield: 68%; and melting point: 195°C–198°C. The FT-IR spectrum (KBr, ν_max_, cm^-1^) shows significant peaks at 3429 (N-H stretching), 3280 (C-H stretching, aromatic), 2946 (C-H stretching, alkyl), 2866 (N-CH_3_ stretching), 1785 (C=N stretching), 1627 (C=O stretching), 1505 (C-C stretching), 1456 (C=C stretching), 1278 (C-O stretching), and 749(C-S stretching). The ^1^H NMR spectrum (400 MHz, DMSO-d_6_, δ, ppm) displays the following signals 9.43 (s, 1H), 8.28 (d, *J* = 8.4 Hz, 1H), 8.08 (d, *J* = 8.3 Hz, 1H), 7.76 (d, *J* = 8.0 Hz, 1H), 7.67–7.38 (m, 2H), 4.60 (s, 1H), 3.66 (t, *J* = 4.6 Hz, 1H), 2.9 (m, 2H), and 1.43 (s, 6H). The ^13^C NMR spectrum (101 MHz, DMSO-d_6_, δ, ppm) reveals peaks at 164.14, 161.98, 154.92, 139.22, 124.68, 121.35, 116.00, 112.31, 106.04, 62.61, 58.57, and 49.55.

##### 2.1.3.8 4-Methoxy-3-(2-morpholinoethoxy)-N-(1,3,4-thiadiazol-2-yl)benzamide (7a)

Compound: C_16_H_20_N_4_O_4_S; molecular weight: 364.42; brown crystalline solid; yield: 74%; and melting point: 225°C–228°C. The FT-IR spectrum (KBr, ν_max_, cm^-1^) shows significant peaks at 3438 (N-H stretching), 3226 (C-H stretching, aromatic), 2936 (C-H stretching, alkyl), 2836 (O-CH_3_ stretching) 1885 (C=N stretching), 1657 (C=O stretching), 1507 (C-C stretching), 1435 (C=C stretching), 1269 (C-O stretching), and 745 (C-S stretching). The ^1^H NMR spectrum (400 MHz, DMSO-d_6_, δ, ppm) displays the following signals 8.28 (s, 1H), 8.08 (d, *J* = 8.3 Hz, 1H), 7.77 (d, *J* = 7.8 Hz, 1H), 7.49 (d, *J* = 7.7 Hz, 1H), 4.92 (s, 1H), 4.61 (t, *J* = 5.7 Hz, 2H), 3.96 (s, 3H), 3.84–3.76 (m, 4H), 3.67 (t, *J* = 4.6 Hz, 2H), and 2.59-2.94 (m, 4H). The ^13^C NMR spectrum (101 MHz, DMSO-d_6_, δ, ppm) reveals peaks at 164.18, 156.25, 156.03, 131.46, 129.75, 123.55, 114.51, 113.29, 109.56, 64.18, 60.77, 55.51, 54.59, and 52.21.

##### 2.1.3.9 4-Methoxy-3-(2-(piperidin-1-yl)ethoxy)-N-(1,3,4-thiadiazol-2-yl)benzamide (7b)

Compound: C_17_H_22_N_4_O_3_S; molecular weight: 362.45; light brown crystalline solid; yield: 72%; and melting point: 228°C–230°C. The FT-IR spectrum (KBr, ν_max_, cm^-1^) shows significant peaks at 3425 (N-H stretching), 3193 (C-H stretching, aromatic), 2981 (C-H stretching, alkyl), 2852 (O-CH_3_ stretching),1765 (C=N stretching), 1684 (C=O stretching), 1527 (C-C stretching), 1442 (C=C stretching), 1278 (C-O stretching) 743 (C-S stretching). The ^1^H NMR spectrum (400 MHz, DMSO-d_6_, δ, ppm) displays the following signals 8.81 (s, 1H), 8.08 (d, *J* = 8.4 Hz, 1H), 7.66 (d, *J* = 7.8 Hz, 1H), 7.49 (d, *J* = 7.7 Hz, 1H), 4.93 (s, 1H), 4.61 (t, *J* = 5.7 Hz, 2H), 4.37 (t, *J* = 5.7 Hz, 2H), 3.96 (s, 3H), 3.67 (t, *J* = 4.6 Hz, 4H), 2.93 (dd, *J* = 6.2 and 4.0 Hz, 4H), and 1.44 (d, *J* = 4.0 Hz, 2H). The ^13^C NMR spectrum (101 MHz, DMSO-d_6_, δ, ppm) reveals peaks at 168.25, 152.82, 147.32, 147.04, 143.78, 127.19, 124.99, 119.76, 112.22, 110.95, 56.68, 56.53, 43.13, 35.16, and 26.37.

##### 2.1.3.10 4-Methoxy-3-(2-(pyrrolidin-1-yl)ethoxy)-N-(1,3,4-thiadiazol-2-yl)benzamide (7c)

Compound: C_16_H_20_N_4_O_3_S; molecular weight: 348.42; dark brown crystalline solid; yield: 71%; and melting point: 252°C–255°C. The FT-IR spectrum (KBr, ν_max_, cm^-1^) shows significant peaks at 3414 (N-H stretching), 3101 (C-H stretching, aromatic), 2935 (C-H stretching, alkyl), 2850 (O-CH_3_ stretching), 1884 (C=N stretching),1675 (C=N stretching), 1510 (C=O stretching), 1448 (C=C stretching),1264 (C-O stretching), and 743 (C-S stretching) The ^1^H NMR spectrum (400 MHz, DMSO-d_6_, δ, ppm) displays the following signals: 8.07 (s, 1H), 7.65 (d, *J* = 7.8 Hz, 1H), 7.50 (d, *J* = 7.8 Hz, 1H), 7.29 (d, *J* = 2.5 Hz, 1H), 4.91 (s, 1H), 4.60 (t, *J* = 5.7 Hz, 2H), 3.96 (s, 3H), 3.66 (t, *J* = 4.6 Hz, 4H), 2.93–2.91 (m, 2H), and 1.43 (d, *J* = 4.0 Hz, 4H). The ^13^C NMR spectrum (101 MHz, DMSO-d_6_, δ, ppm) reveals peaks at 167.68, 165.11, 149.22, 146.48, 143.22, 128.14, 119.19, 116.40, 111.65, 110.38, 55.96, 54.96, 42.56, and 25.81.

##### 2.1.3.11 4-Methoxy-3-(2-(diethylamino)ethoxy)-N-(1,3,4-thiadiazol-2-yl)benzamide (7d)

Compound: C_16_H_22_N_4_O_3_S; molecular weight: 350.44; dark brown crystalline solid; yield: 70%; melting point: 248°C–250°C. The FT-IR spectrum (KBr, ν_max_, cm^-1^) shows significant peaks at 3419 (N-H stretching), 3167 (C-H stretching, aromatic), 2950 (C-H stretching, alkyl), 2849 (O-CH_3_ stretching), 1745 (C=N stretching), 1687 (C=O stretching), 1510 (C-C stretching), 1482 (C=C stretching), 1269 (C-O stretching), 745 (C-S stretching). The ^1^H NMR spectrum (400 MHz, DMSO-d_6_, δ, ppm) displays the following signals: 8.32 (s, 1H), 8.01 (d, *J* = 7.6 Hz, 1H), 7.79 (d, *J* = 7.7 Hz, 1H), 7.51 (d, *J* = 7.8 Hz, 1H), 4.89 (s, 1H), 4.63 (t, *J* = 5.7 Hz, 2H), 3.98 (s, 3H), 3.69 (t, *J* = 4.6 Hz, 2H), 2.95 (dd, *J* = 6.2 and 4.0 Hz, 4H), and 1.24 (t, *J* = 7.2 Hz, 6H). The ^13^C NMR spectrum (101 MHz, DMSO-d_6_, δ, ppm) reveals peaks at 168.25, 152.53, 147.32, 147.04, 143.78, 127.19, 122.60, 116.97, 112.22, 110.95, 56.53, 55.52, 43.13, and 16.54.

##### 2.1.3.12 4-Methoxy-3-(2-(dimethylamino)ethoxy)- N-(1,3,4-thiadiazol-2-yl)benzamide (7e)

Compound: C_14_H_18_N_4_O_3_S; molecular weight: 322.38; reddish brown crystalline solid; yield: 75%; and melting point: 262°C–262°C. The FT-IR spectrum (KBr, ν_max_, cm^-1^) shows significant peaks at 3423 (N-H str.), 3112 (C-H str. Ar.), 2938 (C-H str. Al.), 2865 (O-CH_3_ str.), 2729 (N-CH_3_ str.), 1883 (C=N str), 1664 (C=O str.), 1517 (C-C str.), 1448 (C=C str.), 1286 (C-O str.) 750 (C-S str.). The ^1^H NMR spectrum (400 MHz, DMSO-d_6_, δ, ppm) displays the following signals: 8.04 (s, 1H), 7.65–7.63 (m, 1H), 7.45 (d, *J* = 7.8 Hz, 1H), 7.20 (dd, *J* = 2.7 and 1.1 Hz, 1H), 4.57 (s, 1H), 4.33 (t, *J* = 5.7 Hz, 2H), 3.76 (s, 3H), 2.92–2.84 (m, 2H), and 2.80 (s, 6H). The ^13^C NMR spectrum (101 MHz, DMSO-d_6_, δ, ppm) reveals peaks at 168.20, 160.55, 147.27, 146.99, 143.73, 128.66, 124.94, 116.92, 112.17, 110.90, 56.48, 55.47, and 43.08.

### 2.2 Human carbonic anhydrase (CA II and CA IX) enzyme inhibition assays

Several studies have proven that CAs are intriguing and productive targets for the treatment of epilepsy, despite the fact that their use in this regard was controversial (Zaib et al., 2014; Al-Rashida et al., 2011). Multiple studies have revealed that CAs actively participate in a wide range of illnesses related to the central nervous system (CNS), including epilepsy, and govern multiple physiological functions of the brain (Al-Rashida et al., 2011). Numerous strong connections between CAs and epilepsy have been examined by the study of the impact of extracellular protons, which arise from the CA-mediated hydration of CO_2_, on the N-methyl-D-aspartate (NMDA) receptors, which are triggered during seizures. It has been reported that brain CA activity can cause extracellular acidification, which can inhibit NMDA receptor activity. This could potentially impact epileptic activity by regulating the function of these receptors (Aboul‐Enein et al., 2012b). Moreover, it has been shown that CO_2_ has an anticonvulsant effect and that by blocking CAs, the brain may produce higher-than-usual concentrations of this gas (Rudolph and Knoflach, 2011). Additionally, it has been discovered that CAs control GABAergic processes in the brain, which, in turn, affects seizure activity. Gamma-aminobutyric acid (GABA_A_) receptors are known to be pertinent targets for the treatment of epilepsy because they actively participate in brain activity related to seizures. Numerous investigations suggest that extracellular potassium concentration increases as a result of GABAergic stimulation during seizures.

*In vitro* human carbonic anhydrase II and IX inhibition was carried out using the colorimetric inhibition assay, following the previously reported method with some modifications (Ayati et al., 2016; Kharb et al., 2011). The basic principle involved in this method is based on hydrolyzing p-nitrophenyl acetate to form yellow-colored p-nitrophenol by carbonic anhydrase, which was measured spectrophotometrically (Qamar et al., 2018). In brief, the reaction mixture contained 60 μL of 50 mM Tris-sulfate buffer (pH 7.6, containing 0.1 mM ZnCl_2_),10 μL (0.5 mM) of the test compound in 1% DMSO, and 10 μL of human carbonic anhydrase II and IX (Sino Biological Inc, Beijing, China). All the ingredients were mixed well and pre-incubated at 25°C for 10 min. Substrate p-nitrophenyl acetate was prepared as a 6 mM stock solution using 5% of acetonitrile in buffer and was used fresh. Absorbance was recorded at 348 nm using a microplate reader (Bio Tek Synergy H1 Multimode Microplate Reader, California, United States) to assay the well; approximately 20 μL of the substrate solution was added to attain a 0.6 mM concentration. The total assay volume was adjusted to 100 μL and further incubated at 37°C for 30 min. Furthermore, all the ingredients were mixed properly, and absorbance was measured. Acetazolamide was used as a positive control, while Tris-sulfate buffer was used as a negative control. The results reported are the mean of triplicate experiments (±SEM) and expressed IC_50_ values, which were calculated.

### 2.3 Pharmacological evaluation

#### 2.3.1 Animals

In this study, male Swiss albino mice (20–28 g) were employed. These animals were purchased from LUVAS, Hisar, in the Indian state of Haryana. The experimental procedures were authorized by the Institutional Animal Ethics Committee (IAEC) with the protocol number (BV/IAEC/4062/2020). The mice were housed in groups of six in a regular laboratory environment (25.2 C; 60.2% RH) with free access to food and water (12 h light/dark cycle). The animals were housed, cared for, and handled in compliance with the CPCSEA guidelines. The animals had been exposed to the laboratory environment for 7 days before the experiment.

#### 2.3.2 Drugs

Pentylenetetrazole, sodium valproate, and acetazolamide were purchased from Merck (Merck KGaA, Darmstadt, Germany). The remaining chemicals were obtained from conventional chemical vendors. The solvents utilized were all of analytical grade.

#### 2.3.3 Dose administration

After 7 days of adaptation to laboratory conditions, animals were randomly divided into 13 (I–XIII) experimental groups (each group comprised of six mice). The test compounds and the standard drugs were dissolved in a 0.9% NaCl solution. All the compounds were administered pre-orally to mice 30 min before the induction of convulsion at doses of 30, 100, and 300 mg/kg, with anticonvulsant activity and neurotoxicity assessed at 0.5 and 4 h.

Group I—vehicle-treated group/control group (animals were treated with 300 mg/kg of 0.9% NaCl solution).
Group II and III—standard group (animals were treated with sodium valproate (300 mg/kg) and acetazolamide (100 mg/kg)).
Group IV–XII—test group (animals were treated with synthesized test derivatives 6 (a–e) and 7 (a–e) (30 mg/kg, 100 mg/kg, and 300 mg/kg)).

#### 2.3.4 Acute oral toxicity

The OECD 423 standards were followed while conducting acute oral toxicity tests. The designed synthesized derivatives were orally administered to mice (n = 3) at each dose (2 mg/kg to 500 mg/kg), with only one animal given the dose at a time. The animals were closely monitored for up to 24 h following the dose administration for any signs of toxicity, including excessive activity, convulsions, redness, rashes, drowsiness, overheating, or death.

#### 2.3.5 Neurotoxicity screening

The rotarod test, as described in the literature, was used to evaluate neurotoxicity (NT) in mice. In brief, six mice (one group) were trained to balance on a spinning rod (3 cm diameter and 6 rpm speed) to familiarize with the rotarod apparatus (Orchid Scientific, India). Each mouse underwent three trials to remain on the rotating rod for over 1 minute, with the experiment’s cutoff time (180 s) remaining constant. Different pre-oral (p.o.) doses of test and standard substances were administered to the trained animals. The test compounds were considered neurotoxic at a specific dose level if the trained animal demonstrated a lack of rolling roller performance (i.e., the animals exhibited neurological deficits (sedation and ataxia), as evidenced by their inability to maintain equilibrium on the rod for at least 1 min in each of three trials). The trained animals were evaluated in this manner 30 min after drug administration, and the neurotoxic effect was recorded and compared to those observed in animals given standard drugs (sodium valproate and acetazolamide).

#### 2.3.6 Maximal electroshock‐induced seizure test

**FIGURE 1:**
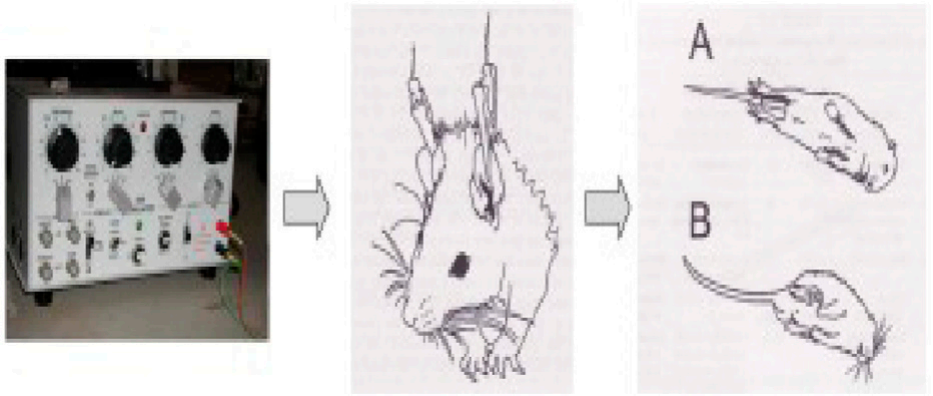
Procedure for the MES method

After half an hour of p.o. administration of the standard and the tested compounds, an electro-convulsometer (Outus, Ambala Cantt, India) delivered an electrical stimulus of sufficient intensity (25 mA, 500 V, 50 Hz, 0.2 s) *via* auricular electrodes to induce maximal seizures (Figure 1). To avoid the animal’s death, a drop of 0.9% saline was injected into the eye before the insertion of the electrodes. The tonic hind limb extension (i.e., the hind limbs of the animals outstretched 180° to the plane of the body axis) served as the basic principle for the occurrence of seizure activity. Abolition of the hind limb tonic extensor (HLTE) component of the seizure is defined as protection. Mice were observed for 60 s for the incidence of HLTE (Hussain et al., 2022).

#### 2.3.7 Subcutaneous pentylenetetrazole (s.c.-PTZ) test

**FIGURE 2:**
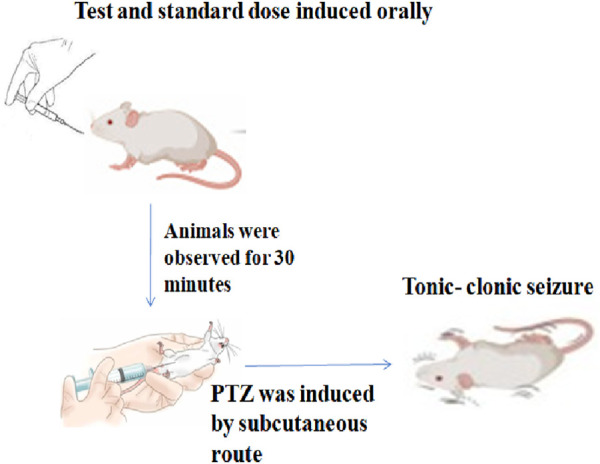
Procedure for the PTZ method.

Thirty minutes after the administration of the test compounds and standard drugs, tonic–clonic convulsions were generated via the subcutaneous (sc) administration of pentylenetetrazole at a dose of 85 mg/kg in 0.9% NaCl solution; following PTZ injection, each mouse was placed separately and monitored for the next 30 minutes for the occurrence of tonic–clonic seizures, which were defined as clonus of the complete body lasting more than 3 s, with an accompanying lack of righting reflex (Figure 2). The period between the first clonus episode and that observed in the control group was recorded and compared to the standard drug. The lack of tonic–clonic convulsions observed during the study period was regarded as the compound’s capacity to protect against PTZ-induced seizures (Porter et al., 1984).

#### 2.3.8 Estimation of oxidative stress and GABA

##### 2.3.8.1 Dissection and homogenization

Brain tissue homogenates were prepared by isolating the brain (tissue) from the mice. Animals were euthanized under anesthesia by ketamine (80 mg/kg) and xylazine (5 mg/kg) after 8 h of inducing test and standard compounds. Mice’s brains (tissue) were quickly removed and weighed, and the whole brain samples were washed twice with ice-cold saline solution before being deposited in glass vials, labeled, and stored in a deep freeze (−30°C) until further use. Brain tissue samples were homogenized with a glass handheld homogenizer (PTFE coated). Tissue homogenates (10% w/v) were prepared in cold 0.1 M phosphate buffer (pH adjusted to 7.4) using a homogenizer. To test biological parameters, the supernatant from homogenates was centrifuged at 12,000 rpm for 20 min at 4°C.

##### 2.3.8.2 Lipid peroxidation

Wills’ (Shimada and Yamagata, 2018) description of chemical reactions to thiobarbituric acid was used to calculate the malondialdehyde (MDA) level, which is a measure of lipid peroxidation (LPO). A measure of 0.5 mL of tissue homogenate (TSH) and 0.5 mL Tris-HCl were mixed, and the resulting mixture was then treated with 10% trichloroacetic acid (1.0 mL) for 2 hours at 37°C. After incubation, the mixture was centrifuged again. The sample tubes were heated for 10 min in a water bath after adding the supernatant and 0.67% thiobarbituric acid. After chilling, 1.0 mL of double-distilled water was added, and absorbance was computed at 532 nm. The reactive chemicals were computed and given as moles of malondialdehyde/mg of protein using a chromophore extinction coefficient (1.56 10^5^M^1^ cm^1^). The results are expressed as nmol MDA/mg protein.

##### 2.3.8.3 Nitrite oxide

Greiss reagent, a solution of 0.5% sulfonamide, 2.5% phosphoric acid, and 0.05% naphthyl ethylenediamine, was used to measure nitric oxide (NO) in TSH. Greiss reagent and TSH absorbance were measured at 540 nm, and the sodium nitrite standard curve was used to calculate the quantity of nitrite (Wills, 1996; Green et al., 1982).

##### 2.3.8.4 Reduced glutathione level

The quantity of glutathione (GSH) in the brain was determined using the Sedlak and Lindsay method (Pant et al., 2023). In brief, brain tissue was deproteinized with an equal amount of 10% TCA and left at 4°C for 2 h. The contents were centrifuged for 15 min at 1200 rpm. The supernatant was mixed with 2 mL of 0.4 M Tris buffer (pH 8.9) containing 0.02 M EDTA (pH 8.9), and then 0.01 M DTNB was added. Finally, the mixture was diluted with 0.5 mL of distilled water to make a total volume of 3 mL, and absorbance was measured using a spectrophotometer at 412 nm, with results expressed as mg GSH/gm tissue.

##### 2.3.8.5 Superoxide dismutase activity

Sun et al.'s approach (Sedlak and Lindsay, 1968) was used to determine Cu, Zn-SOD activity. An oxidase complex (xanthine–xanthine) generates superoxide radicals, which react with NBT to yield the formazan molecule in this process. In brief, a reactive was created by combining 0.1 mM xanthine, 0.1 mM EDTA, 50 mg bovine serum albumin, 25 mM NBT, and 40 mM Na_2_CO_3_ (pH 10.2). To 2.45 mL of reactive, 0.5 mL of TSH was added. Following that, 50 mL of 9.9 nM xanthine oxidase solution was added, the mixture was maintained in a 25°C water bath for 20 minutes, and the reaction was stopped with the addition of 1 mL of CuCl_2_. The sample’s absorbance was measured at 560 nm. TSH was replaced with an equivalent volume of the PBS buffer in the control sample. The quantity of the enzyme that blocked NBT conversion to formazan by 50% was determined as one unit of SOD activity. SOD activity was measured in units per milligram of protein.

##### 2.3.8.6 Measurement of total antioxidant capacity

Total antioxidant capacity (TAC) was measured by mixing 200 μL of tissue samples with 3 mL of ferric-reducing antioxidant power (FRAP) reagent in a vortex. After incubating samples and blank tubes in a water bath for 30 min at 37°C, the absorbance of the samples was measured at 593 nm. For the standard curve, a stock solution of FeSO_4_.7H_2_O in distilled water was produced. Stock solution concentrations were 200, 400, 800, 1200, and 1600 μM. The curve results were given as mM of ferrous sulfate equivalent Fe2+/L (Sun et al., 1988).

##### 2.3.8.7 Estimation of GABA

The supernatant was put on silica gel 60 F_254_ aluminum sheets (10 cm × 10 cm) as the stationary phase, with the mobile phase consisting of water-saturated phenol. Prior to chromatography, TLC plates were prewashed with methanol and activated in an oven at 50°C for 5 min. The 0.1 mL supernatant solutions were applied in the form of bands on TLC plates. At room temperature, ascending development was carried out in a glass chamber saturated with the mobile phase for 30 min until a length of 80 mm was reached. The TLC plates that had been created were air-dried and treated with ninhydrin. Ten formed spots were scraped together and centrifuged with 3 mL of distilled water. The resulting supernatant was spectrophotometrically examined at 570 nm. The method’s specificity was determined by obtaining standard GABA samples and comparing their Rf values. The calibration curve was created by graphing the OD against the concentration (Benzie and Strain, 1999).

### 2.4 Statistical analysis

The outcomes obtained are shown as mean ± SD. One-way analysis of variance (ANOVA) with Tukey’s tests was used to analyze the data. Graph Pad Prism 8.3.0 software was used for all statistical analysis.

### 2.5 Molecular docking

All computational studies were performed on an HP desktop system with an 11th-generation Intel Core processor (2.5 GHz) with Schrodinger 2022–1 Maestro version 13.1.141.

#### 2.5.1 Ligand preparation

The Maestro panel’s 2D sketcher was used to draw all the produced chemicals. They were then transformed into 3D structures in their lowest energy state using the LigPrep module (Huang et al., 2001). The OPLS3e force field, a next-generation force field that spans a significantly wider range of chemical space with greater accuracy (Khan et al., 2016), was used.

#### 2.5.2 Protein preparation and grid generation

The X-ray crystal structures of human carbonic anhydrase II and IX were obtained from the protein data bank (https://doi.org/10.2210/pdb5aml/pdb and https://doi.org/10.2210/pdb5sz5/pdb) using the import option of the protein production wizard in the Maestro panel (Sastry et al., 2013; Roos et al., 2019). The first phase in the protein preparation wizard’s workflow is preprocessing. Missing hydrogen atoms, side chains, or residues were inserted here. It was followed by a review and modification. Chain A was kept in this case, along with hCA II and IX. In the final phase, hydrogen atoms were optimized, water molecules were eliminated, and the protein was optimized using the OPLS3e force field. Using the Glide module’s receptor grid development tool, a receptor grid was produced by selecting CA II and IX as the reference ligands. We chose the binding site of the produced ligand as the active site for our manufactured compounds since we attempted to build CA inhibitors. Van der Waals radii scaling was used to soften the potential for non-polar regions of the receptor, with a scaling factor of 1.0 and a partial charge cutoff of 0.25.

#### 2.5.3 Ligand docking and MM-GBSA calculation

Molecular docking studies were carried out to better understand the binding of the suggested compounds with carbonic anhydrase at the prepared ligand binding site. The docking experiment was carried out in extra precision (XP) mode using the Glide module (Sastry et al., 2013). There are three docking modes in Glide: high-throughput virtual screening (HTVS), standard precision (SP), and enhanced precision (XP). XP is the most accurate method, but it is the most computationally demanding. It can also readily screen out false positives. Van der Waals radii scaling was used to soften the potential for the non-polar portions of the ligands, with a scaling factor of 0.85 and a partial charge cutoff of 0.15. The compounds were rated according to their docking score, and their interactions with the standard were compared. The docking process was confirmed by superimposing the prepared ligand’s docked pose over the co-crystallized pose and determining the root mean square deviation (RMSD). For the docked complexes, MM-GBSA (molecular mechanics, the generalized born model, and solvent accessibility) computations were performed in the Maestro panel using the Prime module (Roos et al., 2019). Ligands were ranked according to their MM-GBSA DG bind scores. The solvent model employed in this investigation was VSGB 2.0 (variable dielectric surface generalized born model).

#### 2.5.4 Drug-likeness analysis

In silico ADMET prediction has reduced compound attrition in later stages of drug discovery (Sastry et al., 2013). As a result, we predicted the ADMET properties of our synthesized compounds using the Maestro panel’s QikProp module. QikProp predicts several relevant physical characteristics and uses them in ADMET calculations. Several metrics were calculated, including the ADME-compliance score (shown by #stars), polar surface area, ionization potential, partition coefficient, absorption, and Lipinski rule violations.

## 3 Results and discussion

### 3.1 Drug design and synthesis

A rational design of the newly synthesized substituted thiadiazole derivatives was achieved on the basis of some previously reported studies. According to the literature, the pharmacophore model for anticonvulsant drugs comprises four binding sites: an aryl hydrophobic binding site (A), a hydrogen bonding domain (HBD), an electron donor–acceptor system (D), and a hydrophobic aryl ring (C).

An aryl hydrophobic binding site (A) consists of an aromatic ring that interacts with hydrophobic regions to help it bind to target proteins that regulate seizures; it interacts with non-polar residues in an enzyme or receptor, contributing to the stability and potency of the chemical because of its hydrophobicity. A hydrogen bonding domain (HBD) contains the -OH or -NH group as these groups have the ability to donate a hydrogen atom in order to create hydrogen bonds with the electronegative atoms of a receptor, such as nitrogen or oxygen, as it increases the pharmacological action and binding affinity of a compound. An electron donor–acceptor system (D) alludes to a component of the molecule that is capable of engaging in electronic interactions, like those with pi-electrons in an aromatic ring or other groups that are rich in electrons. The effectiveness of anticonvulsants may be influenced by this interaction, which can also improve chemical stability and receptor selectivity. A hydrophobic aryl ring (C) targets the protein’s hydrophobic pocket, enhancing the molecule’s overall binding specificity and affinity (Berman et al., 2003).

According to a number of studies, compounds that inhibit CAs may have a synergistic effect when used in combination with traditional AEDs, such as phenytoin, valproate, and acetazolamide. These compounds might enhance the pharmacological activity of AEDs by inhibiting CAs, which could minimize adverse effects or improve seizure control by lowering the dosages of AEDs needed. The well-known hCA inhibitor acetazolamide is already used to treat absence seizures and seizure-related disorders such as febrile seizures in cases of epilepsy. Its action on hCA enzymes contributes to its anticonvulsant properties (Ruffolo et al., 2018; Shukralla et al., 2022).

**FIGURE 3:**
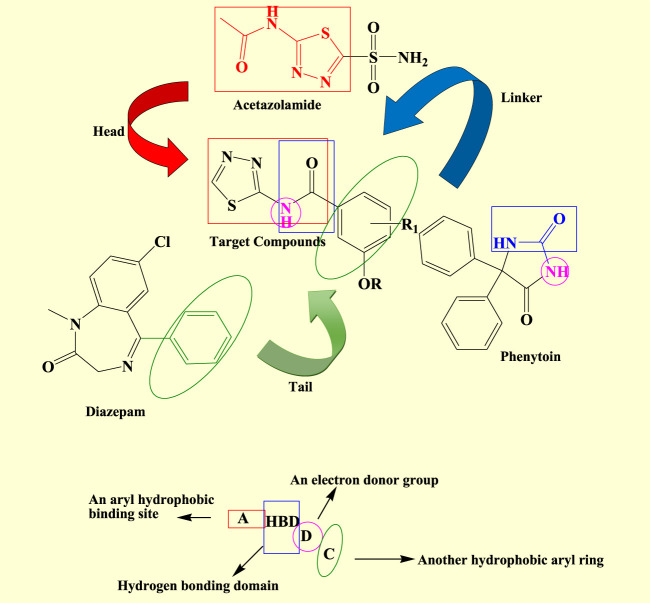
Strategy for designing titled compounds and their pharmacophore patterns.

To develop novel 1,3,4-thiadiazole derivatives as an anticonvulsant agent, the following approaches were used (Figure 3).

• The thiadiazole bioactive nuclei were hybridized to determine their synergistic action (Berman et al., 2003).
• The newly synthesized compounds’ design was influenced by the binding site present in the structures of popular anticonvulsant medications, including acetazolamide, phenytoin, and diazepam (Li et al., 2011).
• The synthesized compound comprises of all four binding sites: an aryl hydrophobic binding site (A), a hydrogen bonding domain (HBD), an electron donor–acceptor system (D), and a hydrophobic aryl ring (C) required for an effective anticonvulsant agent. Substitution with several primary amine groups has been reported to increase anticonvulsant activity in order to improve bioavailability and therapeutic impact, and primary amines are frequently utilized to introduce strong interactions like hydrogen bonds (Berman et al., 2003).

**FIGURE 4:**
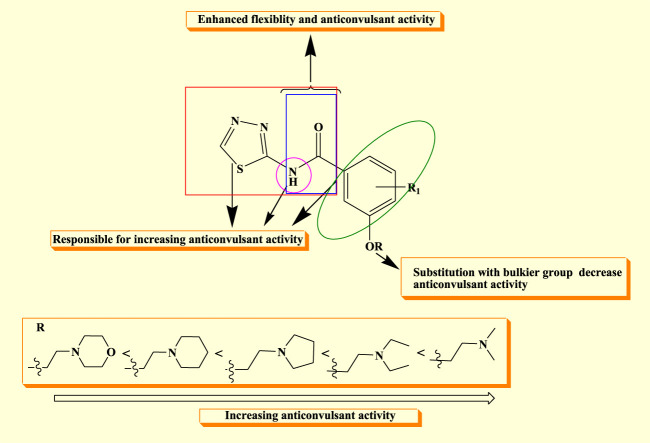
SAR of synthesized derivatives.

The structure–activity relationship of the synthesized compound revealed that semi-carbazones with electron-rich atoms (HBD) linked to the aryl ring (A) enhanced flexibility, which is beneficial for anticonvulsant activity. The linker’s flexibility is considered a significant property for adopting a variety of orientations within the active site, which may yield isoform specific inhibitors. Furthermore, it was observed that substitution with the methyl group at R in the hydrophobic aryl ring (D) increases and prolongs the anticonvulsant activity, while a decrease in activity was observed as the bulkier group was substituted at R. The nitro group (C) has significant activity because it establishes a hydrogen bond with the targeted protein and increases the lipophilicity of the distal aryl ring, which is essential for anticonvulsant activity (Figure 4).

### 3.2 Chemistry

**SCHEME 1:**
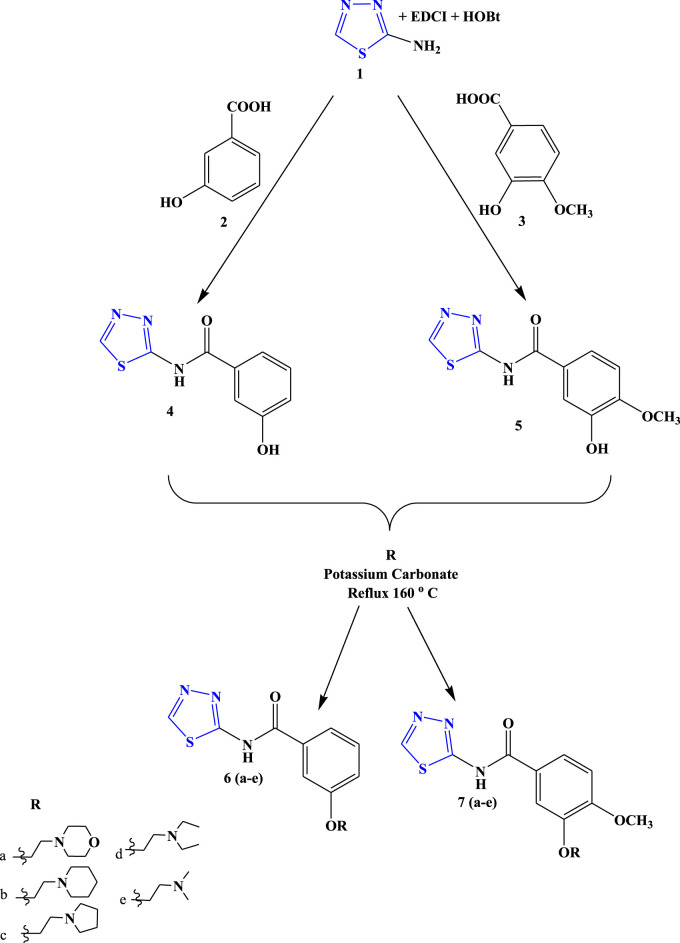
Systematic scheme for designed Novel thiadiazole derivatives.

The target compounds (6 (a–e) and 7 (a–e)) were synthesized through the synthetic route outlined in Scheme 1. 2-Amino-1,3,4-thiadiazole, EDCI, HOBt, and substituted acid were taken as the precursor in DMF, which led the synthesis of compounds 4 and 5. In the next step, a dehydration reaction was carried out using compounds 4 and 5 in the presence of several substituted amines to obtain the final compound (6 (a–e) and 7 (a–e)) in good yields (75%–85%).

### 3.3 *In vitro* human carbonic anhydrase inhibition

The carbonic anhydrase inhibitory activity of novel synthesized 1,3,4-thiadiazole derivatives (**6(a**–**e) and 7(a**–**e)**) toward two physiologically relevant hCA isoforms, CA II and CA IX, was determined using acetazolamide as the standard drug. The inhibitory activity is reported as their IC_50_ ± SEM values in μM in Table 1.

**TABLE 1:** Inhibition data of human carbonic anhydrase (CA) isoforms CA II and CA IX with synthesized compounds **6(a–e)**, **7(a–e)** along with standard (acetazolamide).

Compound	CA II (IC_50_, μM)*	CA IX (IC_50_, μM)*
6a	0.48 ± 0.02	0.40 ± 0.05
6b	0.25 ± 0.03	0.23 ± 0.04
6c	0.38 ± 0.02	0.47 ± 0.02
6d	0.08 ± 0.05	0.10 ± 0.02
6e	0.23 ± 0.02	0.21 ± 0.04
7a	0.97 ± 0.05	0.88 ± 0.04
7b	0.74 ± 0.03	0.73 ± 0.02
7c	0.69 ± 0.02	0.64 ± 0.04
7d	0.48 ± 0.04	0.28 ± 0.03
7e	0.53 ± 0.03	0.61 ± 0.02
Acetazolamide	0.15 ± 0.02	0.12 ± 0.04

*IC_50_ values are calculated as an average of three independent experiments conducted in triplicates.

It was concluded that all the synthesized compounds (**6 (a**–**e)** and **7 (a**–**e)**) exhibited significant-to-good inhibition against epilepsy-related isoforms CA-II and CA-IX in the range of 0.08−0.97 μM and 0.10−0.88 μM, respectively. The synthesized compound showed more inhibition of CA-IX compared to CA-II. Compound **6d** showed the highest inhibition with IC_50_ values of 0.08 ± 0.05 μM (CA-II) and 0.10 ± 0.02 μM (CA-IX), compared to other synthesized compounds, and was almost similar to standard drugs.

### 3.4 Acute oral toxicity

It was observed that no mortality or toxicity signs were observed in the mice up to 500 mg/kg. However, at doses greater than 500 mg/kg, the animals started showing signs of numbness or sedative activity.

### 3.5 Neurotoxicity studies

The term neurotoxicity describes the impairment or dysfunction of the neurological system caused by being exposed to dangerous materials or environmental elements, such as chemicals, medications, or other toxic agents. It can cause a variety of neurological issues by affecting the brain, spinal cord, or peripheral nerves. Analyzing neurotoxicity in animals helps assess possible hazards to human health and offers significant insight into how toxic compounds affect the neurological system. Researchers can determine the neurotoxic impacts of different drugs and comprehend the fundamental pathways of brain destruction by employing behavioral, biochemical, and histological evaluations. These studies are pivotal for the development of safer chemicals, drugs, and environmental policies to protect human health.

The neurotoxicity of all the compounds was evaluated using the rotarod test at three different doses (30 mg/kg, 100 mg/kg, and 300 mg/kg). Neurotoxicity was determined by the inability of the animal to maintain equilibrium on the rod for at least 60 s in each of the three trials. The results obtained in this test demonstrated that none of the investigated compounds induced motor deficits in experimental animals and were safe and non-toxic at the maximum dose (300 mg/kg). The data are presented in Table 2.

**TABLE 2:** Data in the table indicate the minimal dose whereby biological activity was demonstrated in half or more of the mice along with the percentage protection at a particular dose.

Compound	Dose (mg/kg)	Minimum active dose (mg/kg)				% protection				Recovery/death	Neurotoxicity dose (mg/kg)	
		MES		Sc-PTZ		MES		Sc-PTZ				
		0.5 h	4 h	0.5 h	4 h	0.5 h	4 h	0.5 h	4 h		0.5 h	4 h
6a	30	-	-	-	-	-	-	-	-	Recovery	-	-
	100	100	-	-	-	18.46	-	-	-		-	-
	300	-	300	-	300	30.76	43.07	-	19.08		-	-
6b	30	-	-	-	-	-	-	-	-	Recovery	-	-
	100	100	-	-	-	30.76	-	-	-		-	-
	300	-	300	300	300	41.53	50.76	10.20	34.17		-	-
6c	30	-	-	-	-	-	-	-	-	Recovery	-	-
	100	100	100	100	-	26.15	35.38	33.81	-		-	-
	300	-	-	-	300	38.46	46.13	29.26	40.21		-	-
6d	30	30	30	30	30	38.96	41.53	38.59	46.34	Recovery	-	-
	100	-	-	-	-	55.38	60	58.30	66.34		-	-
	300	-	-	-	-	72.30	89.46	70.4	82.88		-	-
6e	30	30	-	30	-	38.46	-	39.02	-	Recovery	-	-
	100	-	100	-	100	32.3	55.38	32.39	63.58		-	-
	300	-	-	-	-	46.15	73.83	46.55	74.2		-	-
7a	30	-	-	-	-	-	-	-	-	Recovery	-	-
	100	100	-	-	-	16.92	-	-	-		-	-
	300	-	300	300	300	-	40	16.64	15.38		-	-
7b	30	-	-	-	-	-	-	-	-	Recovery	-	-
	100	100	-	-	-	29.23	-	-	-		-	-
	300	-	300	300	300	-	47.69	23.07	32.21		-	-
7c	30	-	-	-	-	-	-	-	-	Recovery	-	-
	100	100	100	-	-	24.61	32.30	-	-		-	-
	300	-	-	300	300	35.38	49.23	35.38	36.75		-	-
7d	30	30	30	30	-	30.76	40	42.43	-	Recovery	-	-
	100	-	-	-	100	41.53	63.07	53.27	62.85		-	-
	300	-	-	-	-	61.53	76.92	61.46	75.18		-	-
7e	30	30	-	30	-	36.92	-	34.26	-	Recovery	-	-
	100	-	100	-	100	43.07	58.46	43.07	61.04		-	-
	300	-	-	-	-	60	72.30	60	67.81		-	-
Control	300	-	-	-	-	-	-	-	-	Death	-	-
Sodium valproate	300	300	300	300	300	72.30	63.07	77.72	63.03	Recovery	-	-
Acetazolamide	100	100	100	100	100	66.15	58.46	73.13	58.46	Recovery	-	-

The number of animals tested (n = 6).

The test compounds and standard drugs were suspended in the 0.9% NaCl solution.

Mice were given doses of 30, 100, and 300 mg/kg orally. The table results show the lowest dose at which biological activity was observed in half or more of the mice. The activity of test substances was assessed after 0.5 and 4.0 h of ingestion. The indication - (dash) denotes a lack of action at the maximum dose supplied (300 mg/kg).

MES stands for maximal electroshock method, and sc-PTZ stands for subcutaneous pentylenetetrazole method.

% Protection = (control−test)/(control)∗100.

Reference for the standard drug (De Simone et al., 2013).

### 3.6 *In vivo* anticonvulsant activity

The screening of novel anticonvulsant drugs is mostly based on two well-established tests: the maximal electroshock test (MES) and the subcutaneous pentylenetetrazole test (sc-PTZ). These two tests are being studied as a reliable way to determine the anticonvulsant potential of novel chemical entities. The MES test and sc-PTZ test were performed according to the procedure explained in the material and method on Swiss albino mice to evaluate the anticonvulsant efficacy of synthesized compounds.

### 3.7 Maximal electroshock seizure method

The MES test is a reliable preclinical model for determining the efficacy of experimental medications against generalized tonic–clonic seizures, a form of seizure typically observed in epileptic patients (Dimmock et al., 2000). This test is commonly used for screening novel anticonvulsant drugs and has also shown a clear correlation between the efficacy of new drug molecules in controlling seizures in mice and their efficacy in people against generalized tonic–clonic seizures (Solomon et al., 2009).

Novel synthesized 1,3,4-thiadiazole derivatives were tested for their *in vivo* anticonvulsant activity through this test at the doses of 30, 100, and 300 mg/kg. Sodium valproate and acetazolamide were used as standard drugs to compare the efficacy of novel compounds. Seizure protection was evaluated after 0.5 and 4 h of drug administration, and the results are reported in Table 2.

It was observed that all the synthesized compounds possess moderate-to-good anticonvulsant activity against the MES method but two compounds, **6d** and **7d,** showed the highest protection at low doses. Compound **6d** showed fast onset and long-duration action against MES-generated seizures at a low dose (30 mg/kg) with 38.96% protection after 30 min of drug administration, and the protection capability was found to be better after 4 h, indicating 41.53% protection. The higher dose of 100 mg/kg showed 55.38% and 60% protection, while the 300 mg/kg dose showed 72.30% and 89.46% against MES-induced seizures at time intervals of 0.5 and 4.0 h, while the standard drugs valproic acid (72.30% and 63.07%) and acetazolamide (66.15% and 58.46%) showed protection on the same time intervals, respectively.

### 3.8 Subcutaneous pentylenetetrazole method

Pentylenetetrazole (PTZ), a GABA receptor antagonist, significantly inhibits the GABA_A_ subtype receptor in the brain and works as a powerful chemoconvulsant. It has been well established that the systemic administration of PTZ causes severe generalized myoclonic and absence seizures in rodents. As a result, the PTZ test is used as a reliable paradigm for identifying new anticonvulsant drugs that operate *via* the GABA_A_ subtype receptor (Dimmock et al., 1996; Castel-Branco et al., 2009).

Therefore, synthesized 1,3,4-thiadiazole derivatives were also tested for their *in vivo* anticonvulsant activity in Swiss albino mice through this sc-PTZ method at three different doses, namely, 30, 100, and 300 mg/kg, using sodium valproate and acetazolamide as standard drugs. Seizure protection was evaluated after 0.5 and 4 h of drug administration, and the results are reported in Table 2.

It was noted that compound **6d** with a 30 mg/kg dose showed 38.59% protection after 0.5 h of treatment, while 46.34% protection was observed after 4 h of treatment with the same dose. A dose of 100 mg/kg of this compound has shown 58.30% and 66.34% protection at both time points (0.5 and 4 h), while 70.4% and 82.88% protection were observed with 300 mg/kg of drug administration (0.5 and 4 h). The standard drugs valproic acid (77.72% and 63.03%) and acetazolamide (73.13% and 58.46%) showed protection at the same time intervals.

### 3.9 Estimation of oxidative stress and GABA

**FIGURE 5:**
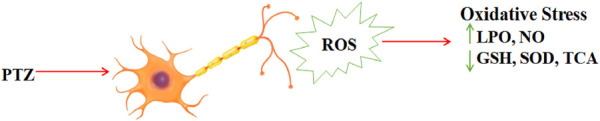
Effect of PTZ on oxidative stress in mice brain.

Oxidative stress in the central nervous system has been observed in various rodent models of experimental epilepsy, including the pilocarpine model, amygdala kindling model, and acute PTZ-induced seizures. A literature survey reveals that PTZ causes oxidative stress, which reduces superoxide dismutase (SOD) and glutathione (GSH) content while increasing MDA and NO levels in brain homogenate. Normally, reactive oxygen species (ROS) are neutralized by a complex antioxidant defense system that includes enzymes like SOD and non-enzymatic antioxidants like GSH (Figure 5). When ROS production is high, the intrinsic antioxidant scavenging capacity is overwhelmed, resulting in oxidative stress, which can cause tissue injury and may activate apoptotic processes. In the present study, the levels of lipid peroxidation (MDA), NO, GSH, SOD, and TAC were estimated to evaluate the effect of the two most potent compounds (**6d** and **7d**) against the oxidative stress processes induced by PTZ injection, taking sodium valproate as the standard according to the procedure explained in *Material and Methods*.

#### 3.9.1 Estimation of MDA levels

**FIGURE 6:**
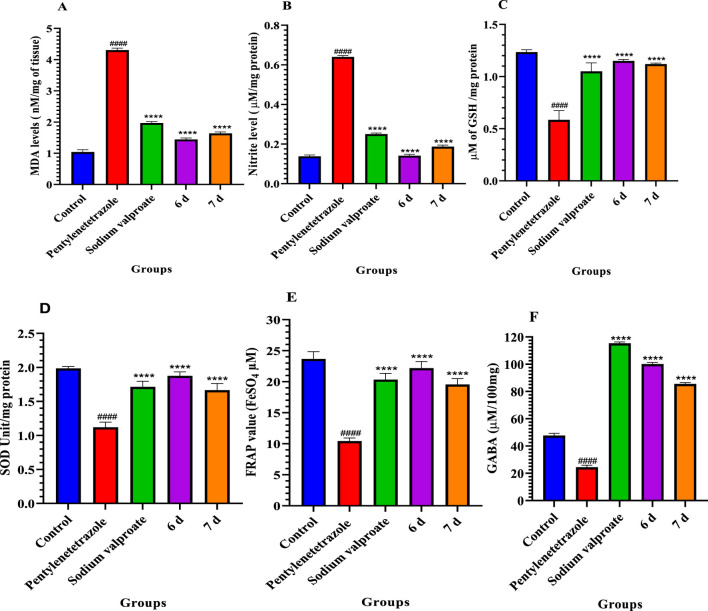
Effect of compounds **6d** and **7d** lipid peroxidation **(A)**, nitrite level **(B)**, glutathione content **(C)**, superoxide dismutase **(D)**, total antioxidant capacity **(E)**, and GABA level **(F)** compared to the pentylenetetrazole-treated group of mice. Values are expressed as the mean ± SD, n = 6. ####p < 0.0001 (positive control is compared with the normal control) and ****p < 0.0001 (treatment groups are compared with the positive control).

LPO in the brain can be induced by many chemical compounds and brain injuries such as epilepsy (Huang et al., 2001). MDA is one of the major aldehydes produced by membrane LPO products and can be used as an LPO marker. LPO products are more likely to harm the brain than other tissues. Furthermore, LPO is an indicator of irreversible neuronal cell membrane phospholipid damage and has been proposed as a probable cause of epileptic activity (Loscher et al., 1991; Freitas et al., 2005; Pazdernik et al., 2001; Dal-Pizzol et al., 2000). Pentylenetetrazole significantly increased lipid peroxidation and decreased antioxidant activity; the current investigation suggests an increase in oxidative stress. The MDA level significantly reversed in the case of mice treated with these two compounds, indicating reduced lipid peroxidation (Figure 6A).

#### 3.9.2 Estimation of NO level

Increased NO levels in the brain can impair cognitive function. The production of nitric oxide was determined by the accumulation of nitrate in the brain supernatants. In this study, we have observed that compounds **6d** and **7d** significantly reduced NO levels compared to the pentylenetetrazole-treated group (Figure 6B).

#### 3.9.3 Estimation of GSH activity

GSH is a key cellular antioxidant that protects cells from oxidative damage. In this study, the pentylenetetrazole group remarkably decreased the GSH level compared to the normal control. However, the administration of compounds **6d** and **7d** exhibited a significant increase in GSH levels compared to the pentylenetetrazole-treated group (Figure 6C).

#### 3.9.4 Estimation of SOD activity

SOD is an antioxidant enzyme that plays a crucial role in detoxifying superoxide anions. SOD levels in brain homogenate were significantly decreased in the PTZ-administered group. However, treatment with compound **6d** showed a significant increase in SOD activity (Figure 6D).

#### 3.9.5 Estimation of total antioxidant capacity

The FRAP assay was used to assess the antioxidant effect of non-enzymatic defense in **6d-** and **7d**-treated mice. It was observed that compounds **6d** and **7d** exhibited a significant increase in TAC levels compared to the pentylenetetrazole-treated group (Figure 6E).

#### 3.9.6 Estimation of GABA

The effect of compounds **6d** and **7d** on GABA levels in the brain was measured spectrophotometrically after 2 h of drug administration (30 mg/kg, p.o). It was observed that compounds **6d** and **7d** exhibited a significant increase in GABA levels compared to the pentylenetetrazole-treated group (Figure 6F).

### 3.10 Molecular docking

**FIGURE 7:**
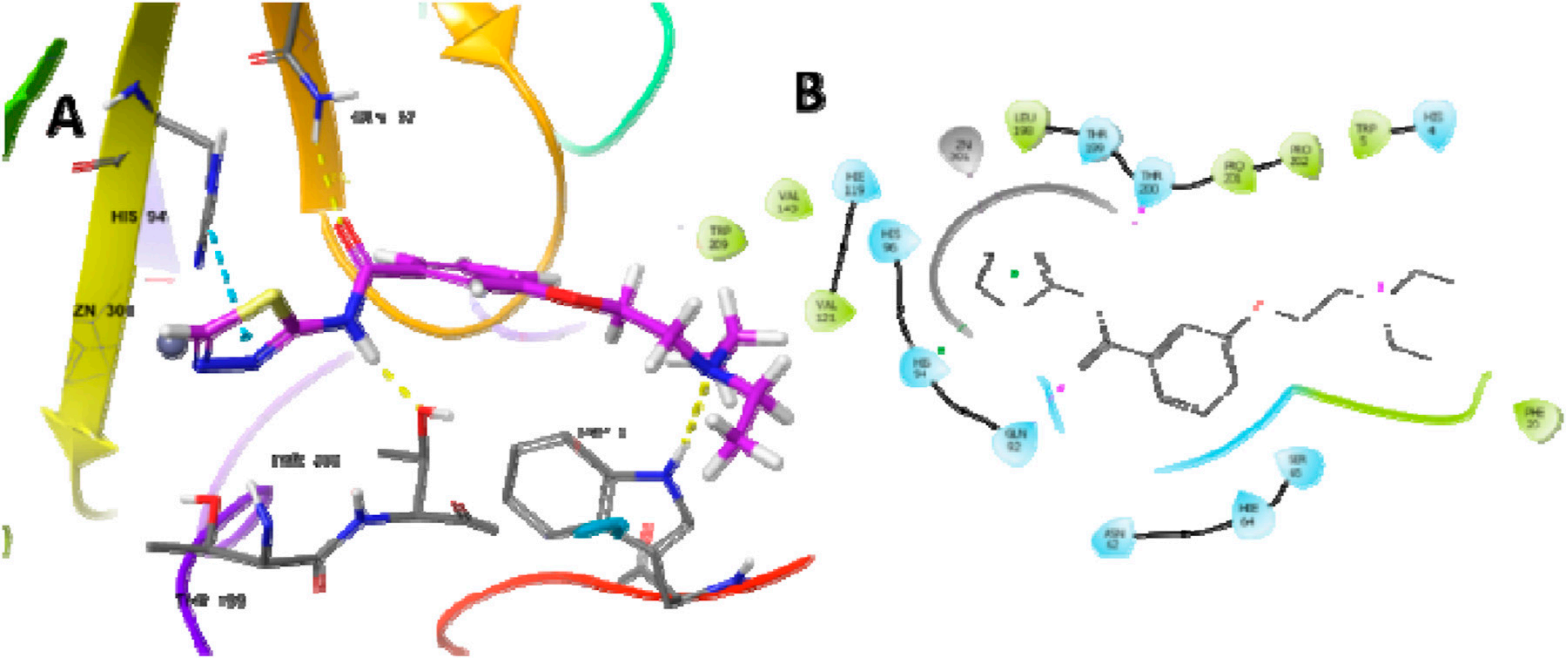
Docking studies of compound **6d** into active site human carbonic anhydrase IX (PDB-ID-5SZ5) **(A)** 3D docking model and **(B)** 2D docking model.

**FIGURE 8:**
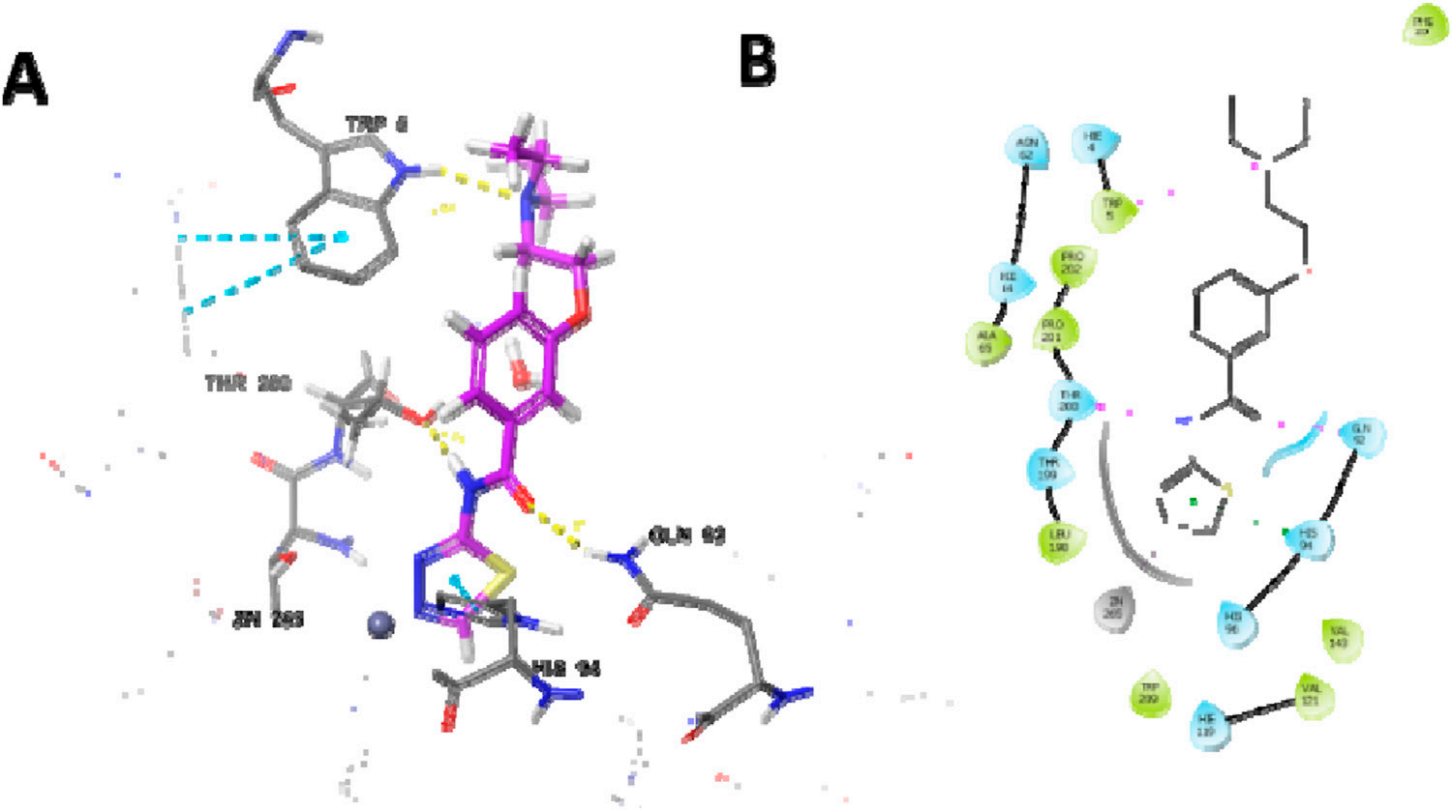
Docking studies of compound **6d** into active site human carbonic anhydrase II (PDB-ID-5AML) **(A)** 3D docking model and **(B)** 2D docking mode.

The most potent compound was docked into the active site of human carbonic anhydrase (hCA) IX and II (PDB-ID 5SZ5 and 5AML) to comprehend the possible binding interactions on the active site. In PDB-ID-5SZ5, compound **6d** showed very good interaction on the active site with a docking score of −6.032, closer to the co-crystal, whereas in PDB-ID-5AML, it showed a docking score of −4.288. In PDB-ID-5SZ5, we observed that the thiadiazole ring of compound **6d** interacted with zinc metal through the co-ordination bond and showed hydrophobic interactions through pi–pi stacking with HIS94. The nitrogen atom of the amide group showed hydrogen bonding with THR200 at a distance of 1.71 Å, and the oxygen atom of the carbonyl group showed hydrogen bonding with GLN92 at a distance of 2.06 Å. Tertiary nitrogen of compound **6d** interacted with TRP5 through hydrogen bonding at a distance of 2.32 Å (Figure 7). In 5AML, compound **6d** showed hydrogen bonding between the NH of the amide group and THR200 with a distance of 1.72 Å. The carbonyl group of compound **6d** interacted with GLN92 through hydrogen bonding with a distance of 2.07 Å. The thiadiazole ring of compound **6d** showed metallic zinc bonding through the metal co-ordination bond. It also showed hydrogen bonding between tertiary nitrogen and TRP5 with a distance of 2.48 Å (Figure 8). The docking score of the most potent compound is mentioned in Table 3.

**TABLE 3:** Docking score of compound **6d** along with the co-crystal and the standard drugs on hCA IX and II (PDB-ID 5SZ5 and 5AML).

S.N.O	Title	PDB-ID-5SZ5	PDB-ID-5SAML
1	6d	−6.032	−4.288
2	Sodium valproate	−3.604	−4.071
3	Acetazolamide	−2.405	−2.93
4	Prepared ligand (co-crystal)	−6.154	−6.969

#### 3.10.1 *In silico* prediction of the ADME profile of compound 6d

In the present study, compound **6d,** which is the most promising compound based on *in vitro* and *in vivo* results, was exposed to molecular property prediction and drug-likeness, and the results are shown in Table 4.

**TABLE 4:** Predicted ADME parameters of compound **6d**.

S.N.O	ADME parameter	Maximum range or recommended values	Compound 6d
1	Molecular weight	<500	320.409
2	Rule of five (number of violations of Lipinski’s rule of five)	4	0
3	PSA (van der Waals surface area of polar nitrogen and oxygen atoms)	7.0–200.0	77.517
4	SASA (total solvent accessible surface area in square angstroms)	300–1000	637.507
5	Dipole moment	1.0–12.5	9.584
6	Molecular volume (total solvent accessible volume)	500–2000	1081
7	Donor H.B.	0–6	1
8	Acceptor H.B.	2–20	7.25
9	QPlogKhsa (prediction of binding to human serum albumin)	−1.5–1.5	−0.213
10	QPlogPo/w (predicted octanol/water partition coefficient	−2.0–6.5	2.194
11	QPpolrz (predicted polarizability in cubic angstroms)	13.0–70.0	33.941
12	QPlogBB (predicted brain/blood partition coefficient)	−3.0–1.2	−0.521
13	QPlogKp (predicted skin permeability)	−8.0–1.0	−4.102
14	QPPCaco (predicted apparent Caco-2 cell permeability in nm/sec)	<25 is poor, >500 is great	228.905
15	P.O.A. (predicted human oral absorption on 0%–100% scale)	<25% is poor, >80% is high	82.025%

## 4 Conclusion

Since human carbonic anhydrase isoforms regulate pH balance, ion transport, and neuronal excitability in the central nervous system, inhibiting these enzymes is a feasible treatment approach for epilepsy as it improves the brain’s GABAergic inhibition, which assists with controlling seizures. CA inhibition has the potential to alter the extracellular space’s pH, especially by decreasing it (making it more acidic). In order to reduce excessive neuronal firing and manage seizures, this acidic environment may increase GABA-A receptor activation and GABAergic inhibition.

A series of substituted 1,3,4‐thiadiazole derivatives (**6(a–e) and 7(a–e)**) were synthesized as effective CA inhibitors with medium-to-high micro-molar inhibitory activity on isoforms involved in convulsions to explore prospective of new anticonvulsant agents. The novel inhibitors are made up of heterocyclic sulfonamide as a zinc-binding motif, which is coupled to various heteroaryl tails *via* hydrogen binding domains as linkers. Compounds **6d** and **7d** were the most powerful CA-II and CA-IX inhibitors, with good-to-excellent anticonvulsant activity in MES and sc-PTZ seizure models with long-duration action at minimum dose, with excellent antioxidant activity and significant increase in GABA levels compared to the pentylenetetrazole-treated group. Furthermore, neurotoxicity was determined by the rotarod method, and none of them showed significant acute neurological toxicity (no impairment in motor coordination and balance was observed). It was observed that the derivatives containing the di-ethyl methyl group in their structure showed the highest anticonvulsant activity compared to other synthesized derivatives and standards. This suggests that di-ethyl methyl groups, along with the binding site, plays a major role in enhancing the potency of the compound as a anticonvulsant agent. Thus, the study identified the synthesized derivative compound **6d**, which showed an IC_50_ value of 0.08 ± 0.05 μM and 0.10 ± 0.02 μM against CA-II and CA-IX, with 72.30% and 89.46% protection against MES-induced seizures, and 70.4% and 82.88% protection against PTZ-induced seizures at time intervals of 0.5 and 4.0 h at 300 mg/kg. It was found to be the most powerful, safe, and effective anticonvulsant agent, working by inhibiting the CA receptor, and can be exploited to produce beneficial therapeutic medicines for the treatment of certain types of epilepsy.

## Data Availability

The raw data supporting the conclusions of this article will be made available by the authors on request.
