# Supplementary material for: Design, synthesis, and in vitro, in vivo, and in silico evaluation of novel substituted 1,3,4-thiadiazole derivatives as anticonvulsant agents
Source: Front Chem. 2025 Feb 12;12:1515866. doi: 10.3389/fchem.2024.1515866 (PMC11861159; doi:10.3389/fchem.2024.1515866)

Supplementary Information

Design, synthesis, in-vitro, in-vivo, and in-silico evaluation of novel substituted 1,3,4-thiadiazole derivatives as anticonvulsant agents

Tulika Anthwal[1], Swati Pant[1], Preeti Rana[2], Sumitra Nain[1]*

1. Department of Pharmacy, Banasthali Vidyapith, Banasthali, Rajasthan, India, 304022
2. National Institute of Pharmaceutical Education and Research (NIPER), Balanagar, Hyderabad, Telangana, India

***Correspondance**: Dr. Sumitra Nain, Department of Pharmacy, Banasthali Vidyapith, Banasthli, Rajasthan, India, 304022.
Email: nainsumitra@gmail.com

Contents:

1. Docking score of all the synthesized compounds along with the co-crystal and the standard drugs on hCA IX and II (PDB-ID 5SZ5 and 5AML): **Table 1**

2. FT-IR and NMR (1H and 13C) Spectra of all the synthesized compounds.

Table 1: Docking score of all the synthesized compounds along with the co-crystal and the standard drugs on hCA IX and II (PDB-ID 5SZ5 and 5AML).

S. No	Title	Docking score	
		**5AML**	**5SZ5**
1	6d	-5.036	-6.297
2	6c	-4.904	-5.993
3	7c	-5.496	-5.789
4	7d	-5.368	-5.444
5	6e	-4.548	-5.442
6	7e	-4.550	-5.348
7	6a	-4.989	-5.069
8	7a	-4.889	-5.061
9	6b	-5.269	-4.994
10	7b	-3.601	-4.892
11	Sodium valproate	-4.071	-3.604
12	Acetazolamide	-2.93	-2.405
13	Preparedligand (Cocrystal)	-6.969	-6.154

1. 4-hydroxy-N-(1,3,4-thiadiazol-2-yl)benzamide (4)

FT-IR Data

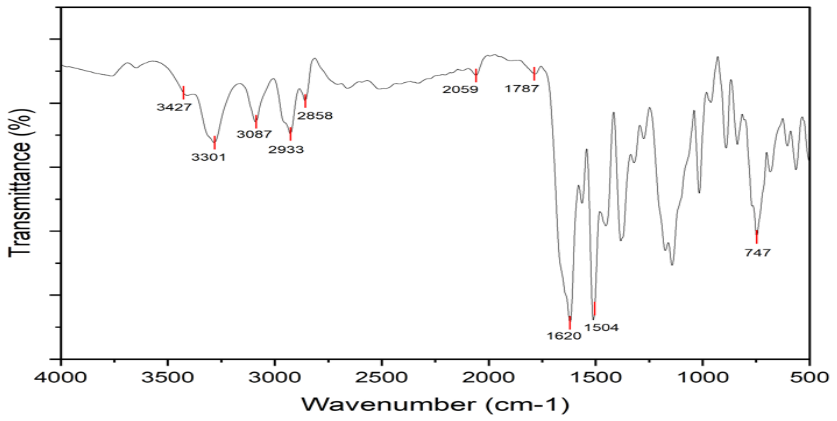

NMR Data

^{1}H

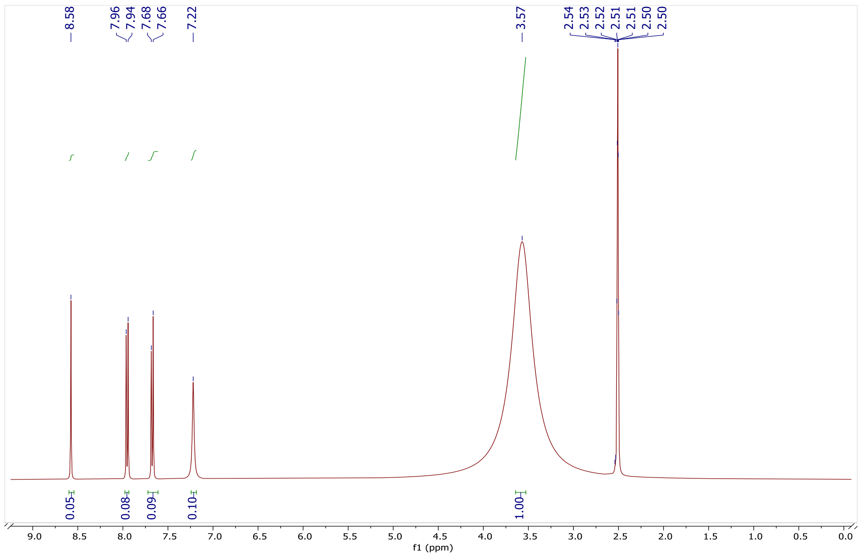

^{13}C

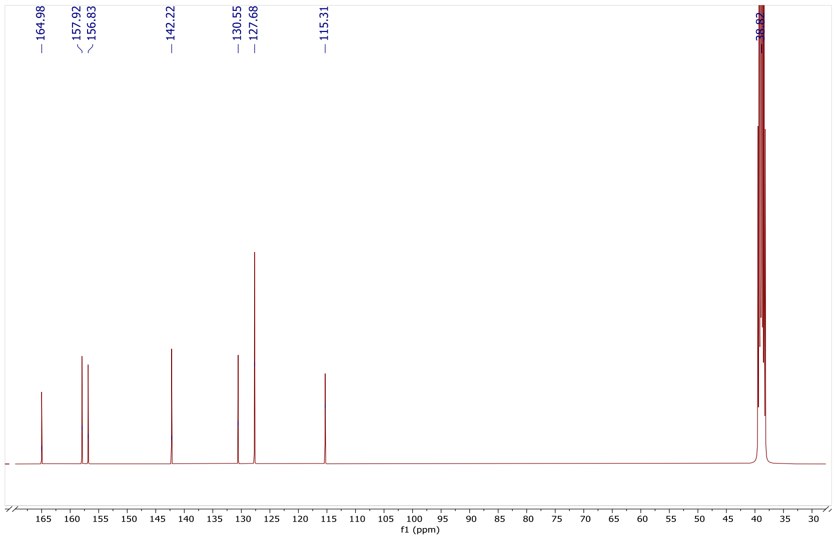

2. 4-hydroxy-3-methoxy-N-(1,3,4-thiadiazol-2-yl)benzamide (5)

FT-IR Data

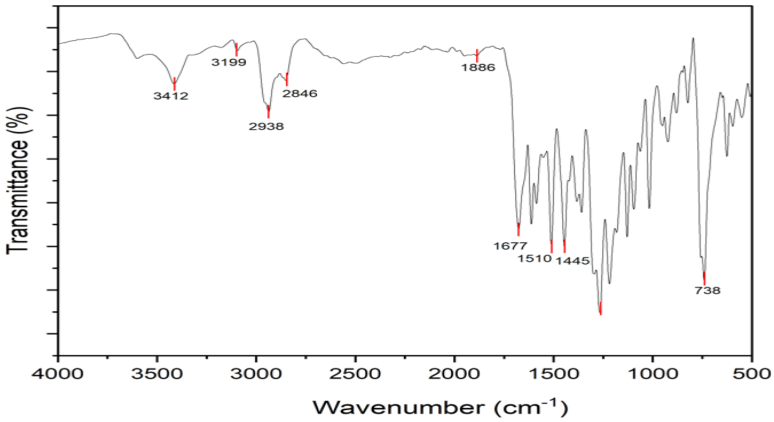

NMR Data

^{1}H

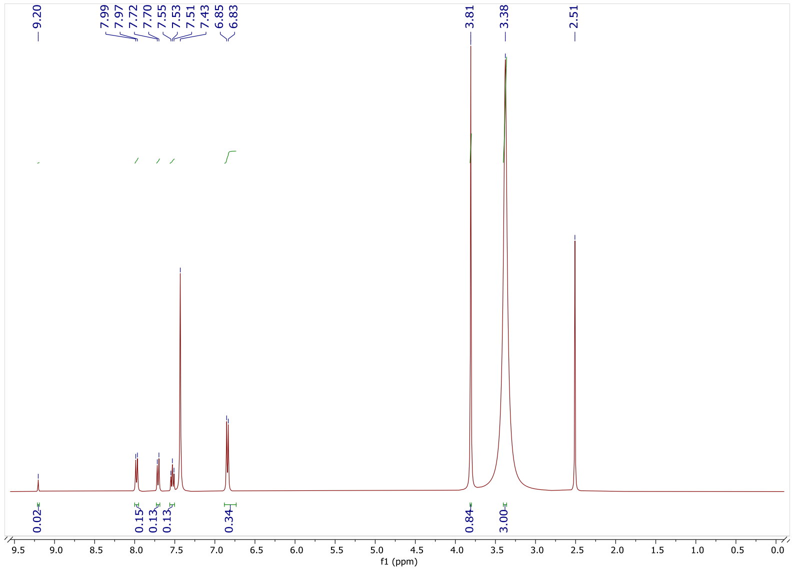

^{13}C

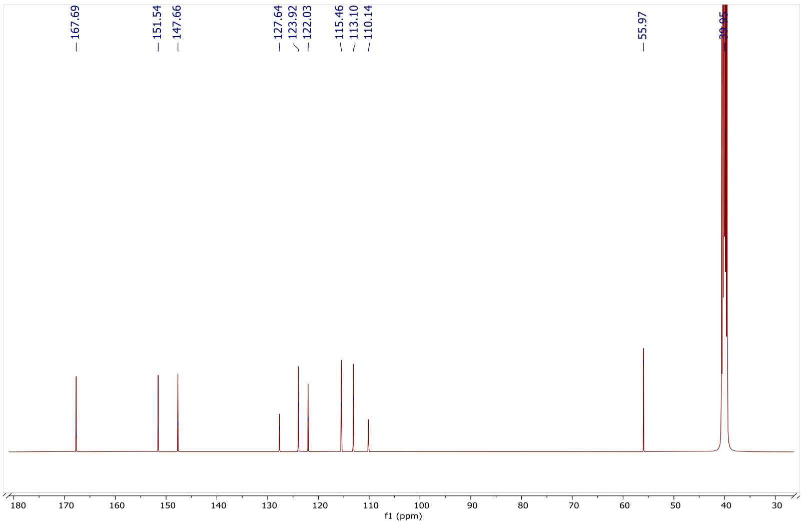

3. 4-(2-morpholinoethoxy)-N-(1,3,4-thiadiazol-2-yl)benzamide

FT-IR Data

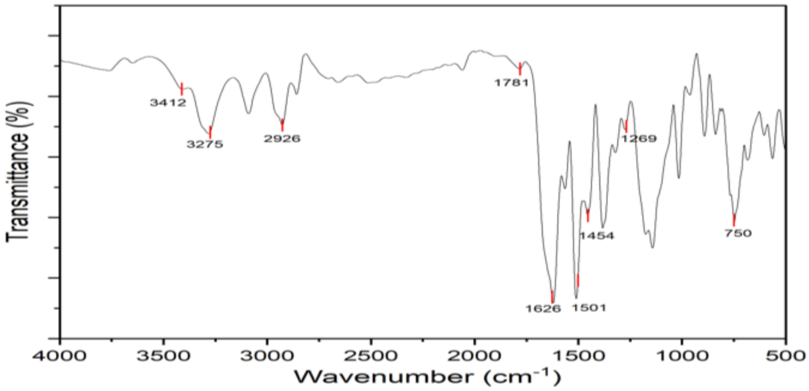

NMR Data

[1]H

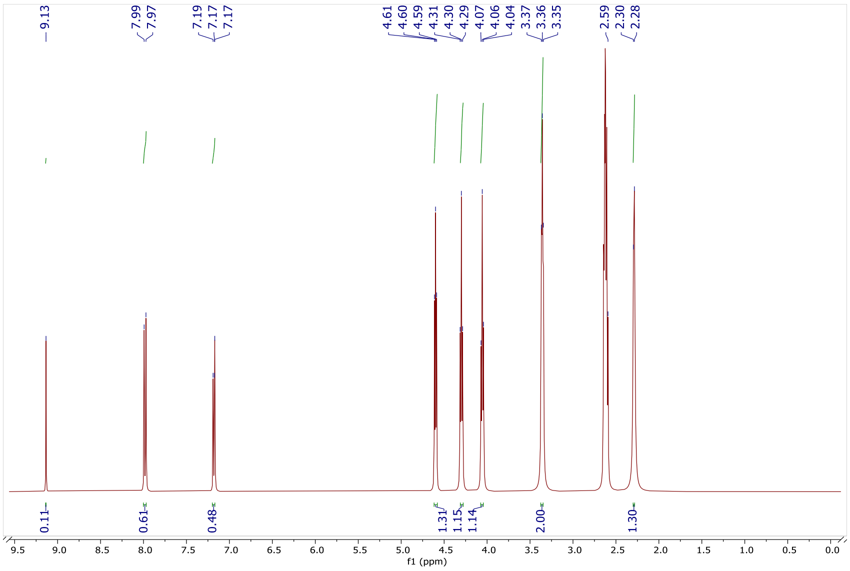

[13] C

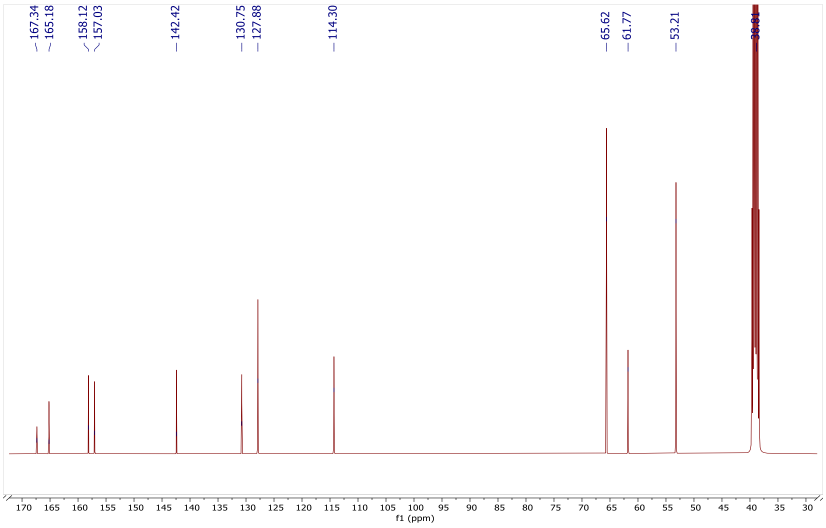

4. 3-methoxy-4-(2-(piperidin-1-yl)ethoxy)-N-(1,3,4-thiadiazol-2-yl)benzamide (6b)
FT-IR Data

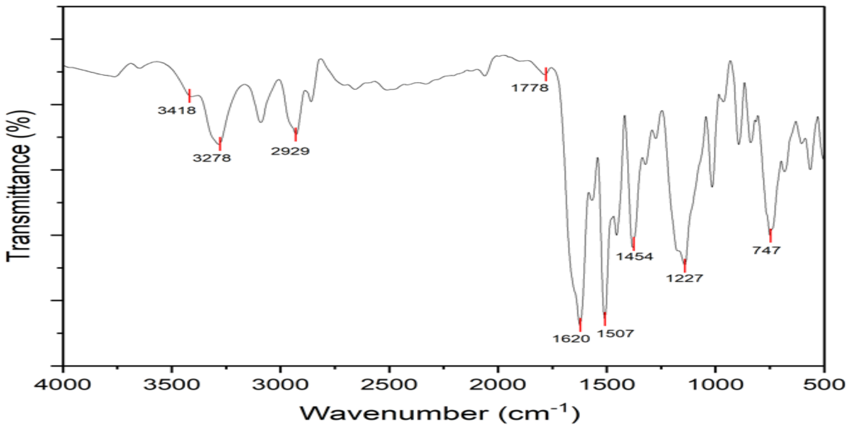

NMR Data

^{1}H

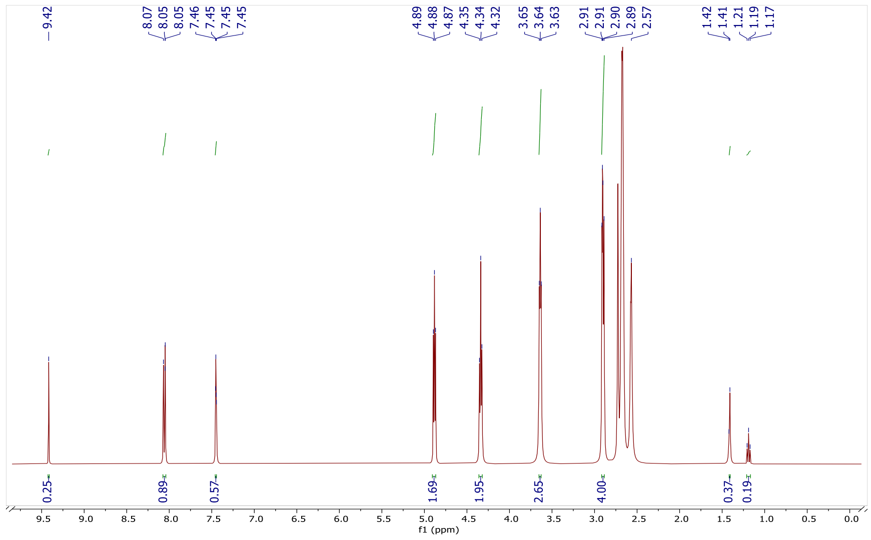

^{13} C

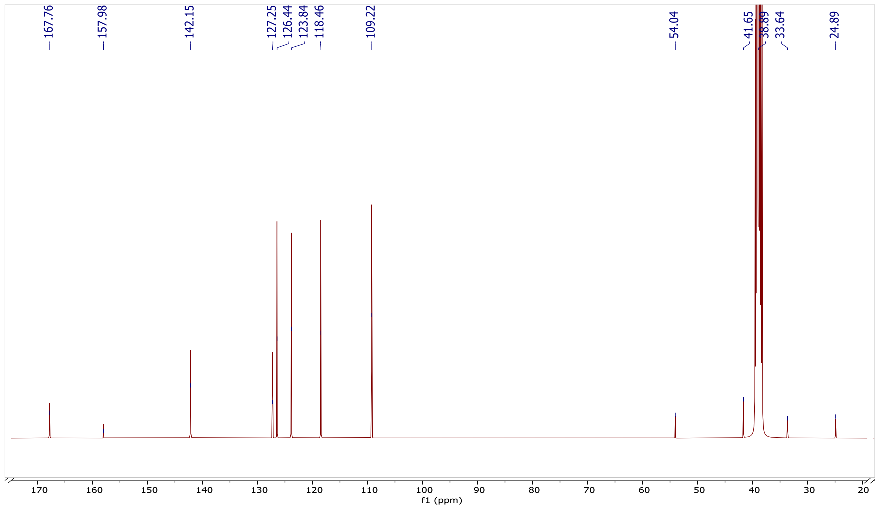

5. 3-methoxy-4-(2-(pyrrolidin-1-yl)ethoxy)-N-(1,3,4-thiadiazol-2-yl)benzamide (6c)

FT-IR Data

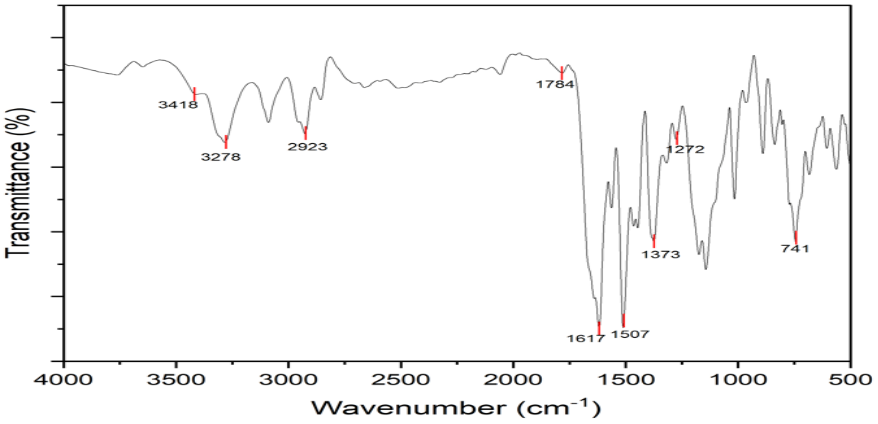

NMR Data

^{1}**H**

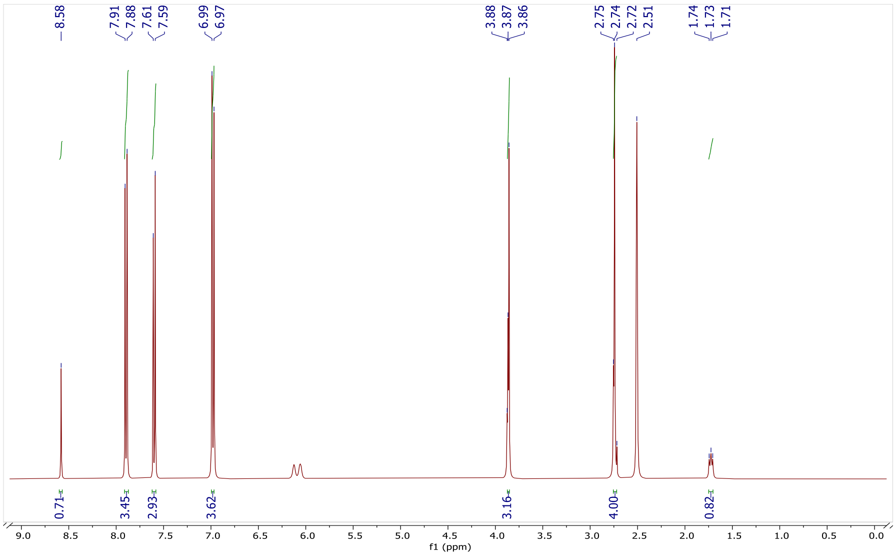

^{13} **C**

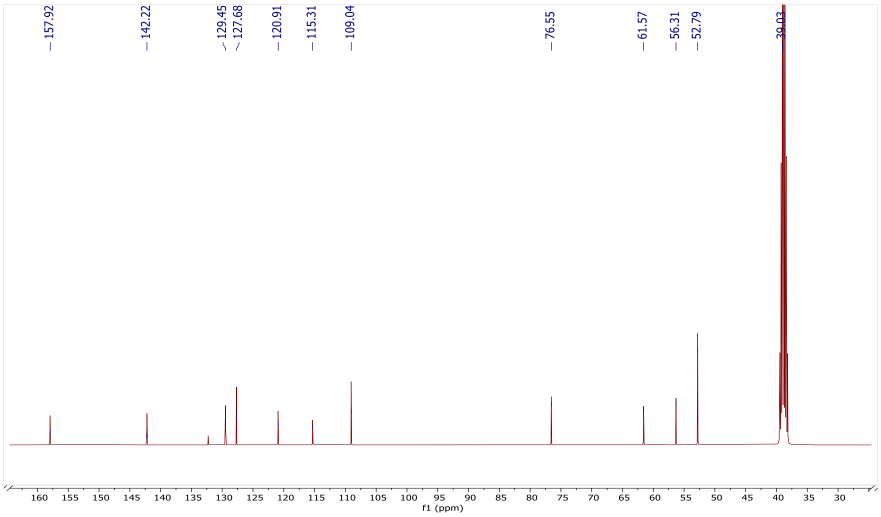

6. 4-(2-(dimethylamino)ethoxy)-3-methoxy-N-(1,3,4-thiadiazol-2-yl)benzamide (6d)

FT-IR Data

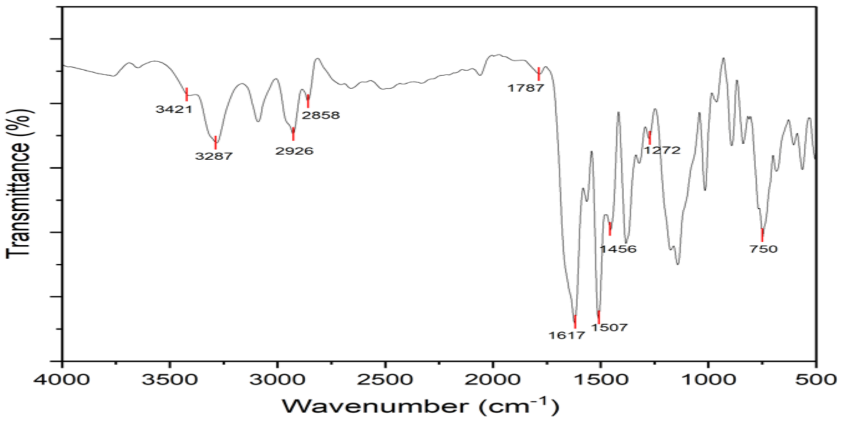

NMR Data

^{1}H

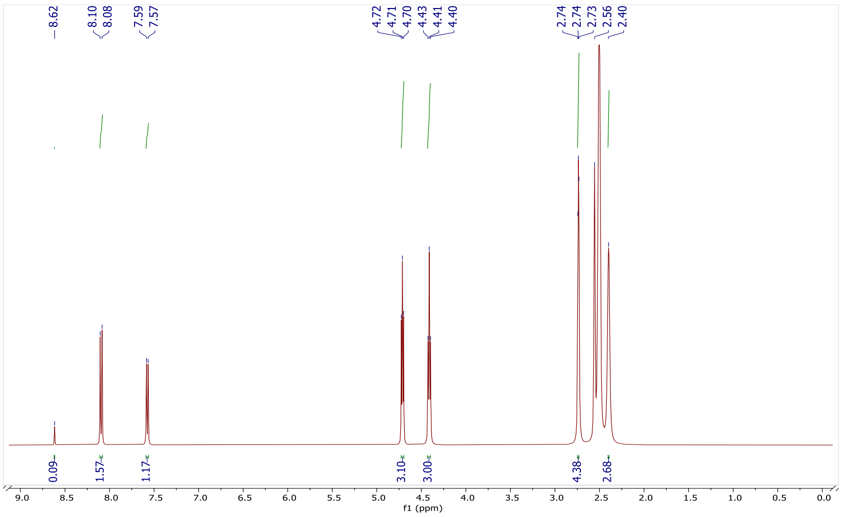

^{13}C

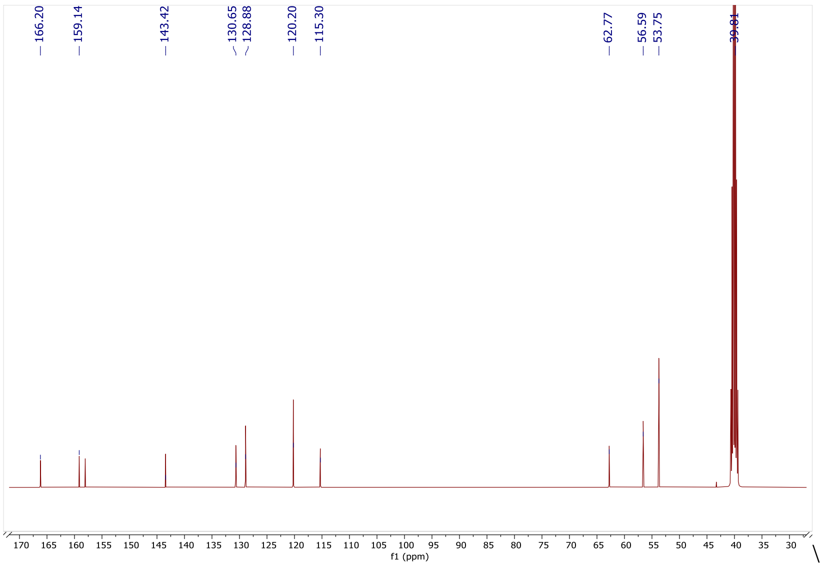

7. 4-(2-(diethylamino)ethoxy)-N-(1,3,4-thiadiazol-2-yl)benzamide (6e)

FT-IR Data

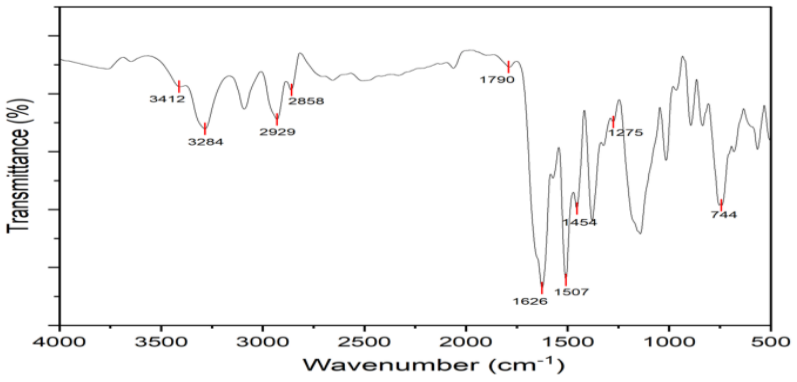

NMR Data

^{1}H

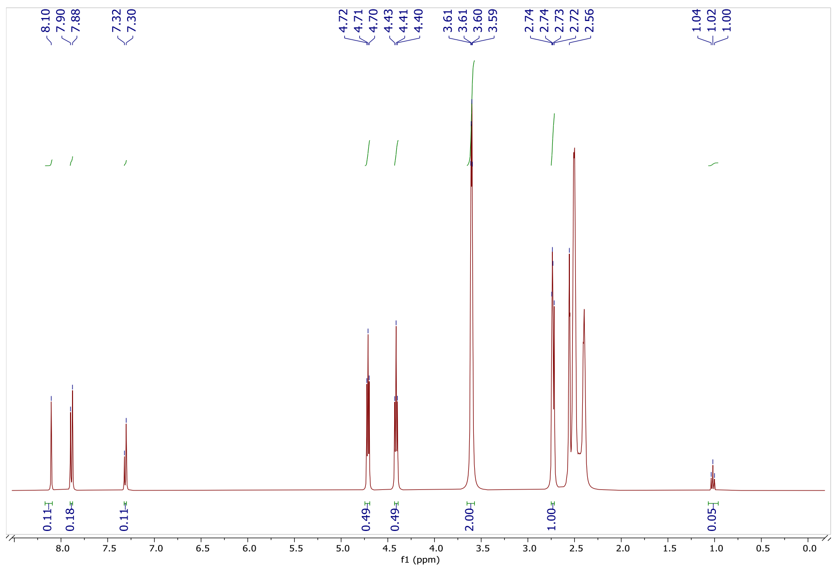

^{13}C

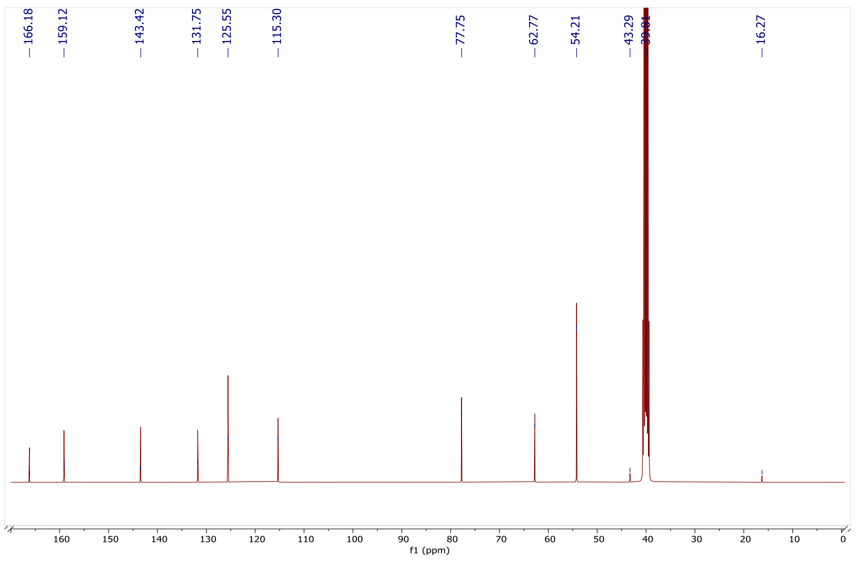

8. 3-methoxy-4-(2-morpholinoethoxy)-N-(1,3,4-thiadiazol-2-yl)benzamide (7a)

FT-IR Data

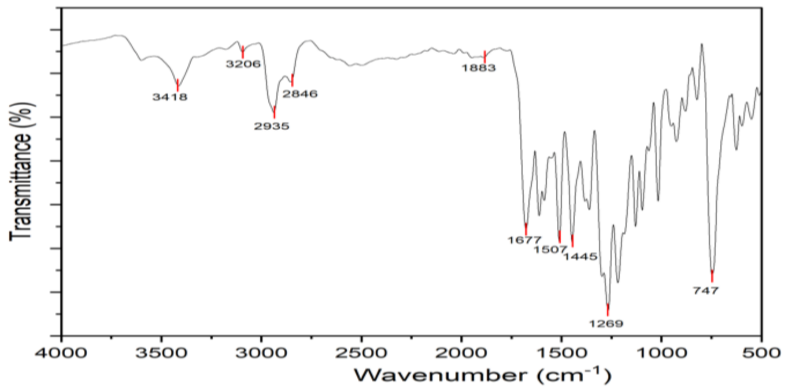

NMR Data

^{1}H

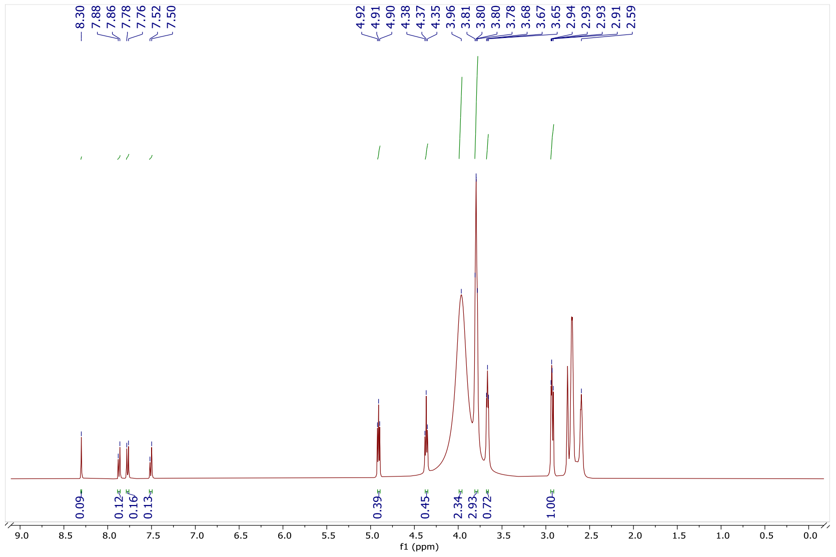

^{13}C

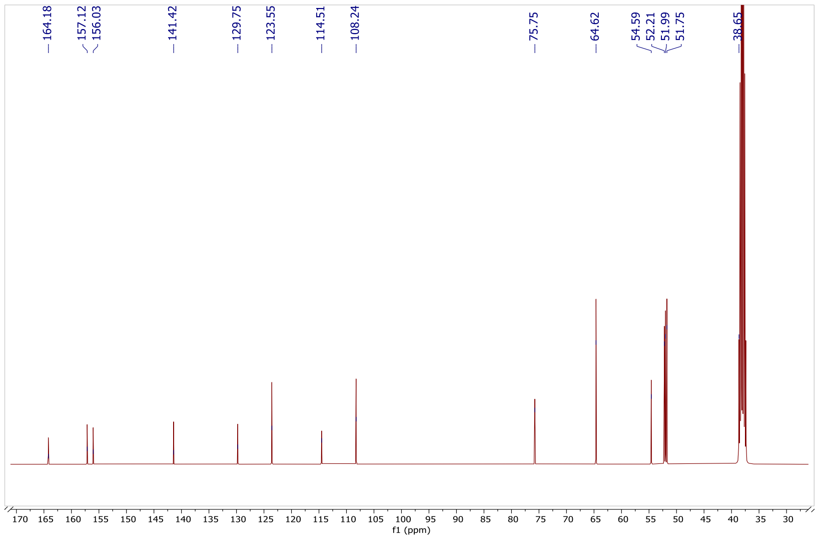

9. 3-methoxy-4-(2-(piperidin-1-yl)ethoxy)-N-(1,3,4-thiadiazol-2-yl)benzamide (7b)

FT-IR Data

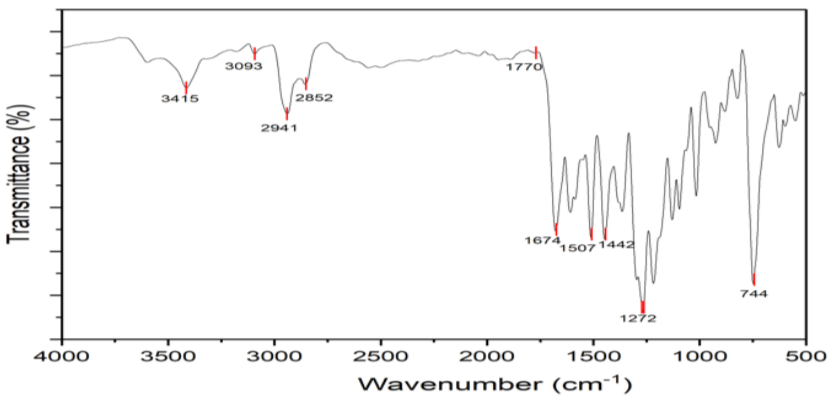

NMR Data

^{1}H

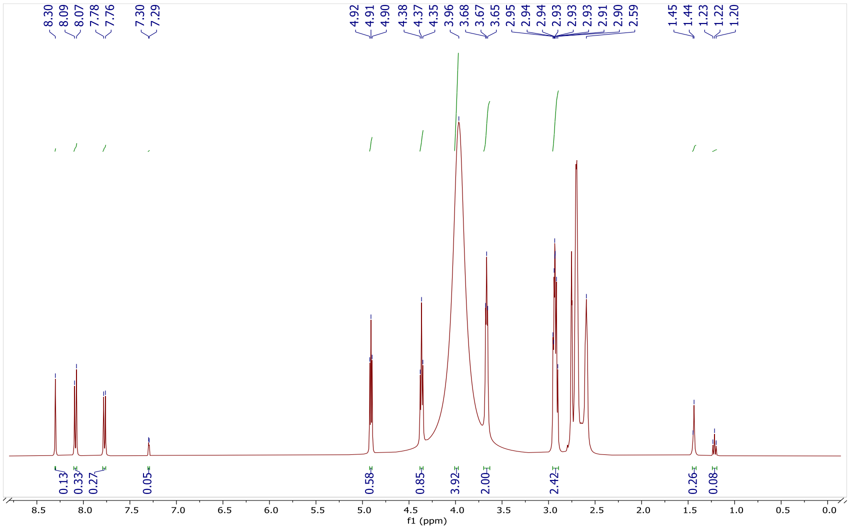

^{13}C

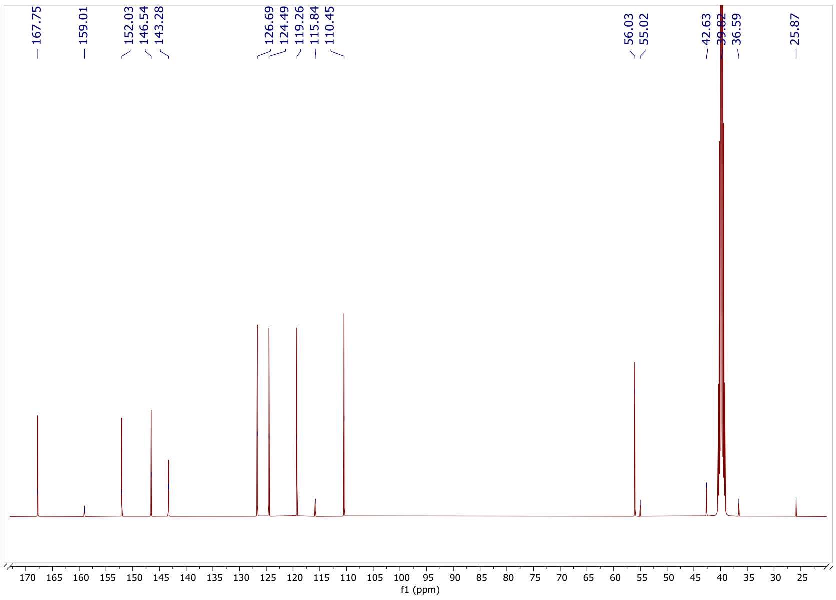

10. 3-methoxy-4-(2-(pyrrolidin-1-yl)ethoxy)-N-(1,3,4-thiadiazol-2-yl)benzamide (7c)

FT-IR Data

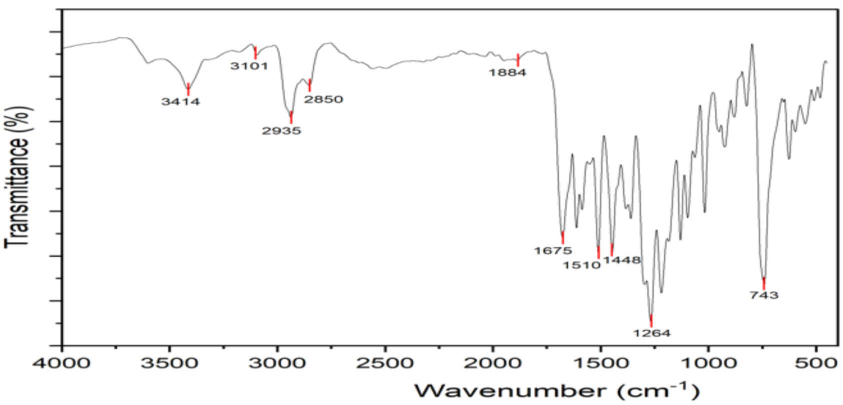

NMR Data

^{1}**H**

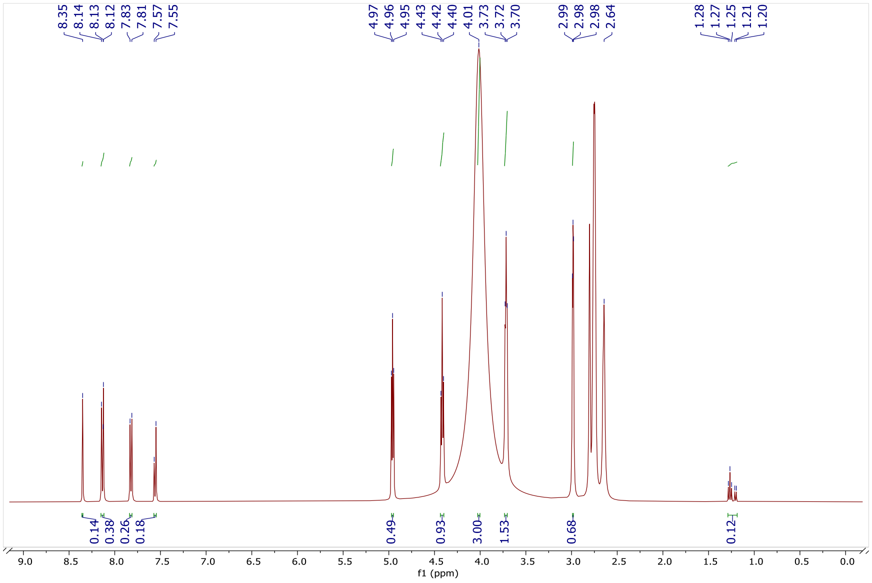

^{13}**C**

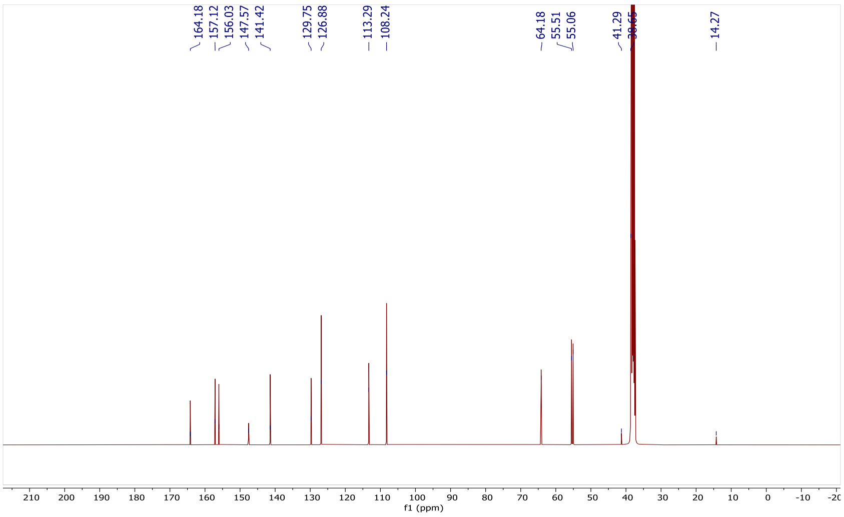

11. 4-(2-(dimethylamino)ethoxy)-3-methoxy-N-(1,3,4-thiadiazol-2-yl)benzamide (7d)

FT-IR Data

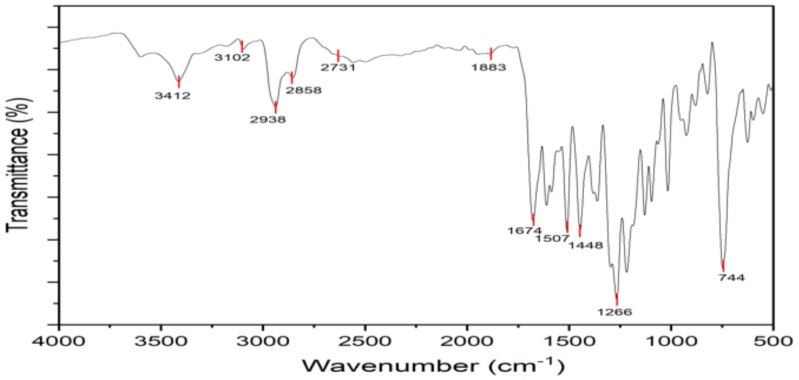

NMR Data

^{1}H

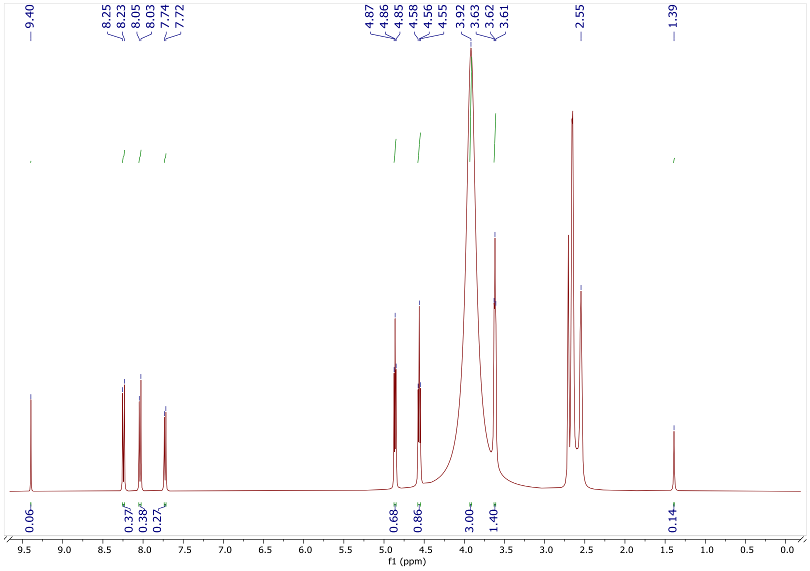

^{13} C

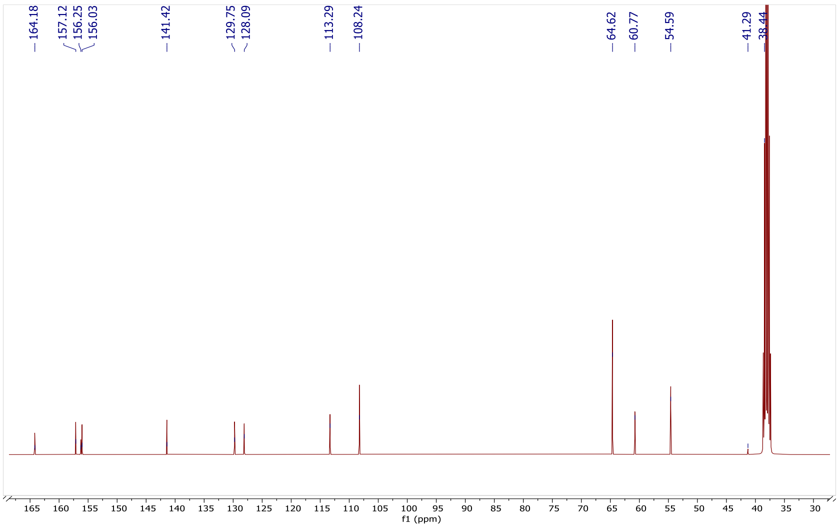

12. 4-(2-(diethylamino)ethoxy)-3-methoxy-N-(1,3,4-thiadiazol-2-yl)benzamide (7e)

FT-IR Data

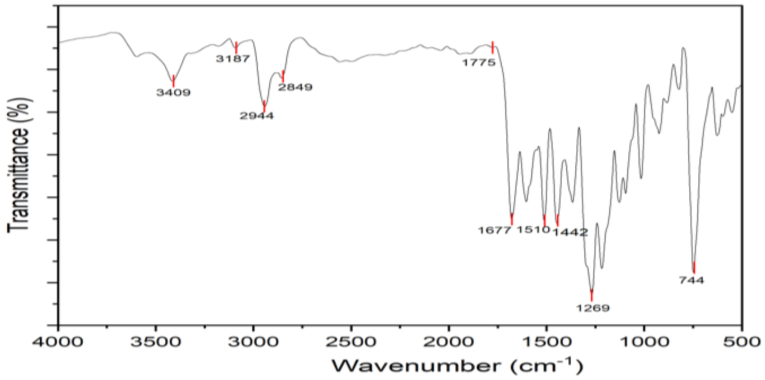

NMR Data

^{1}H

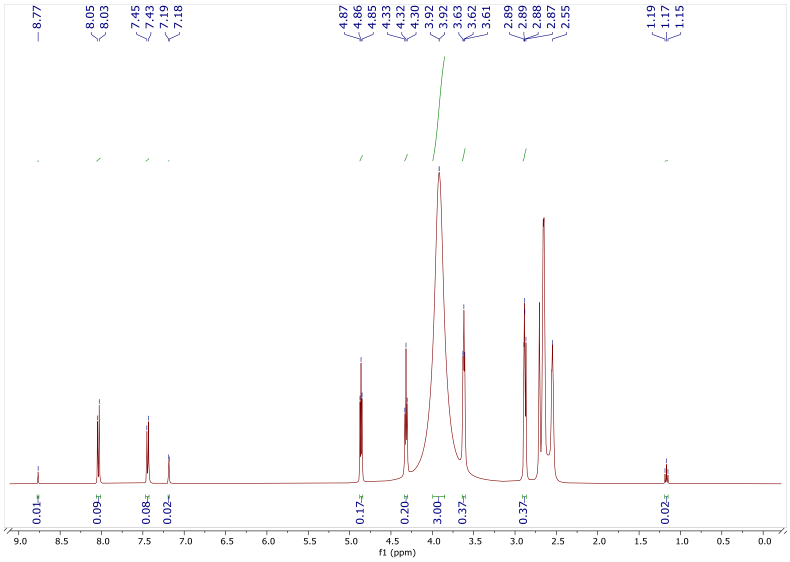

^{13}C

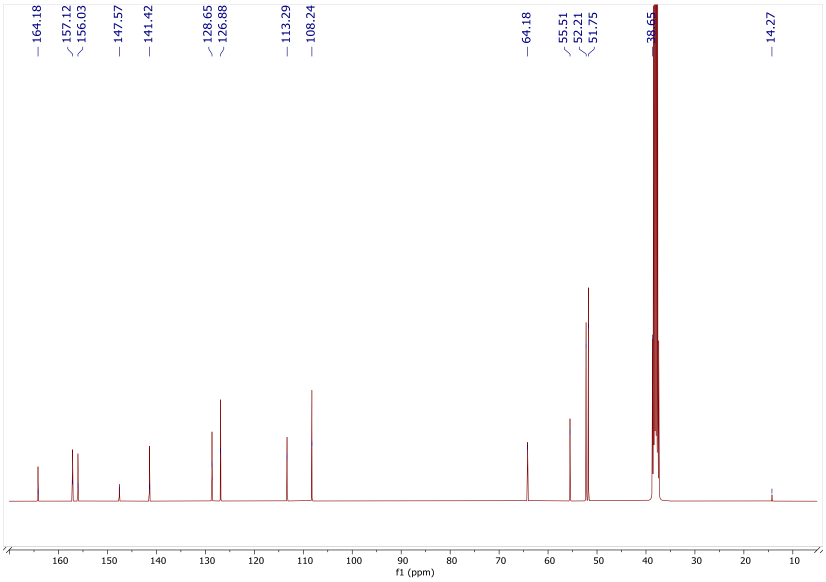

Supplement: Supplementary file 1 [file DataSheet2.pdf]
