# Supplementary material for: Design, synthesis, and in vitro, in vivo, and in silico evaluation of novel substituted 1,3,4-thiadiazole derivatives as anticonvulsant agents
Source: Front Chem. 2025 Feb 12;12:1515866. doi: 10.3389/fchem.2024.1515866 (PMC11861159; doi:10.3389/fchem.2024.1515866)

Supplementary Information

Design, synthesis, in-vitro, in-vivo, and in-silico evaluation of novel substituted 1,3,4-thiadiazole derivatives as anticonvulsant agents

Tulika Anthwal[1], Swati Pant[1], Preeti Rana[2], Sumitra Nain[1]*

1. Department of Pharmacy, Banasthali Vidyapith, Banasthali, Rajasthan, India, 304022
2. National Institute of Pharmaceutical Education and Research (NIPER), Balanagar, Hyderabad, Telangana, India

***Correspondance**: Dr. Sumitra Nain, Department of Pharmacy, Banasthali Vidyapith, Banasthli, Rajasthan, India, 304022.
Email: nainsumitra@gmail.com

Contents:

1. Docking score of all the synthesized compounds along with the co-crystal and the standard drugs on hCA IX and II (PDB-ID 5SZ5 and 5AML): **Table 1**

2. FT-IR and NMR (1H and 13C) Spectra of all the synthesized compounds.

Table 1: Docking score of all the synthesized compounds along with the co-crystal and the standard drugs on hCA IX and II (PDB-ID 5SZ5 and 5AML).

S. No	Title	Docking score	
		**5AML**	**5SZ5**
1	6a	-4.372	-3.638
2	6b	-3.448	-3.431
3	6c	-3.41	-4.059
4	6d	-3.03	-4.238
5	6e	-3.483	-3431
6	7a	-3.53	-4.636
7	7b	-3.993	-4.353
8	7c	-3.6	-4.572
9	7d	-4.523	-3.876
10	e	-4.296	-4.023
11	Sodium valproate	-4.071	-3.604
12	Acetazolamide	-2.93	-2.405
13	Preparedligand (Cocrystal)	-6.969	-6.154

Spectral data

4

FT-IR

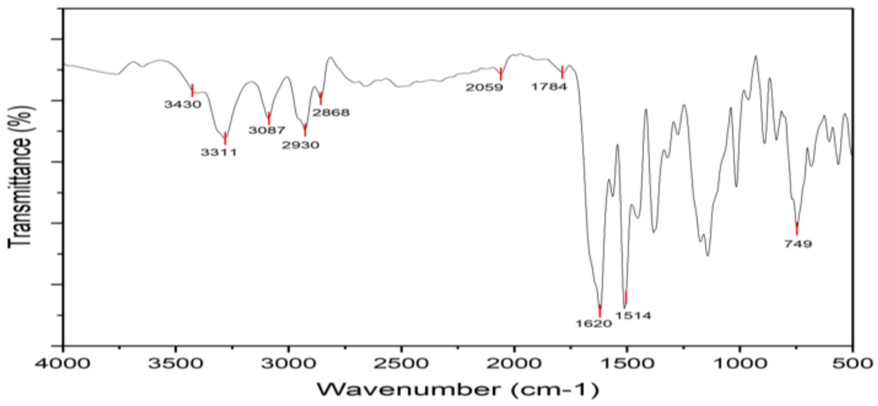

^{1}HNMR

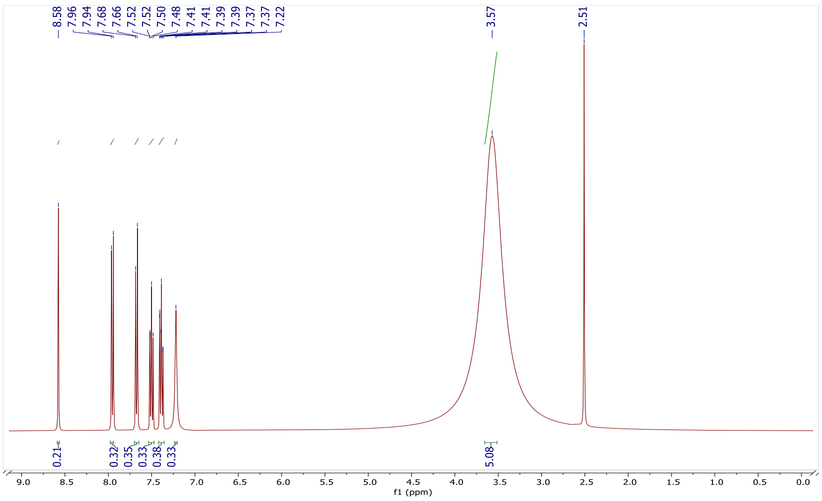

^{13} CNMR

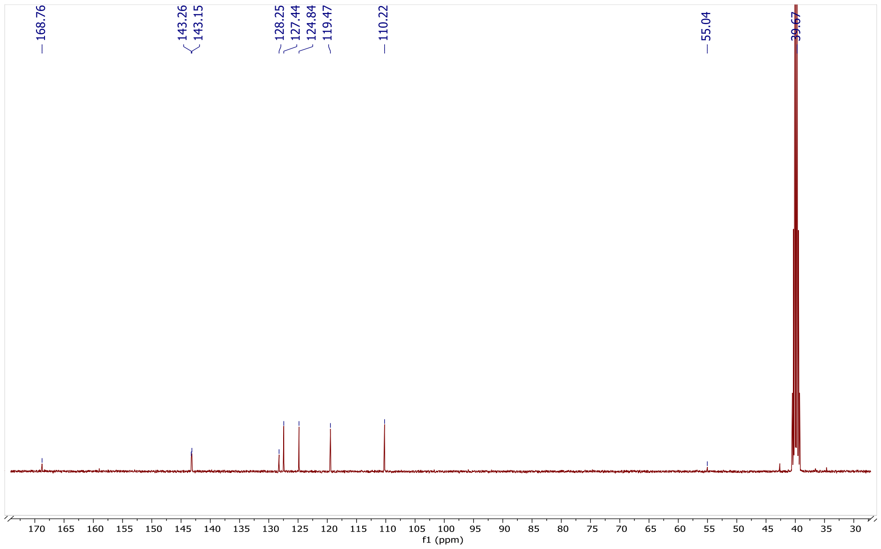

5

FT-IR

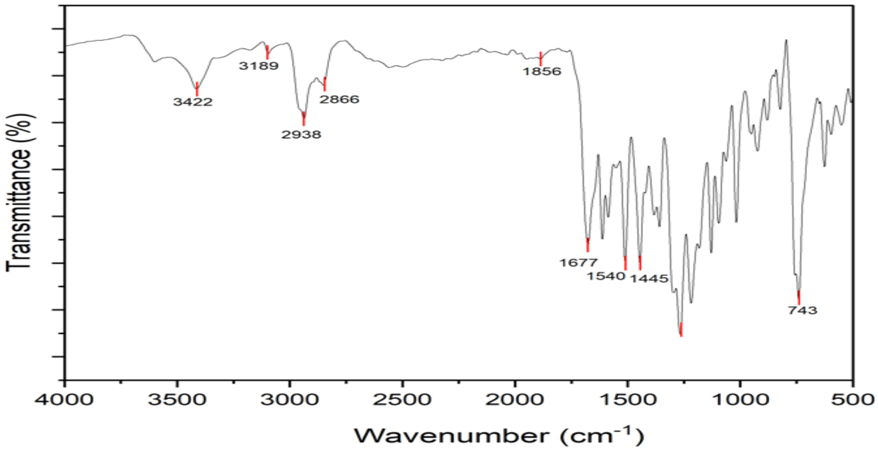

^{1} HNMR

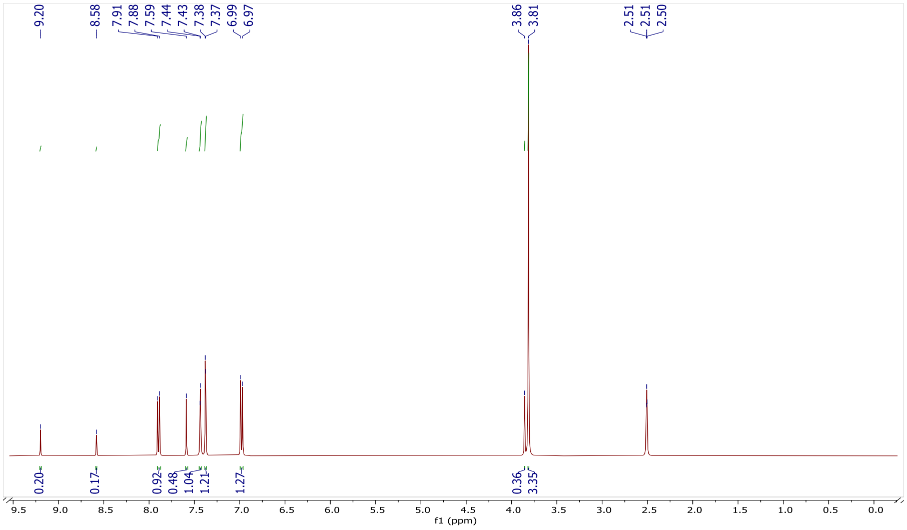

^{13} CNMR

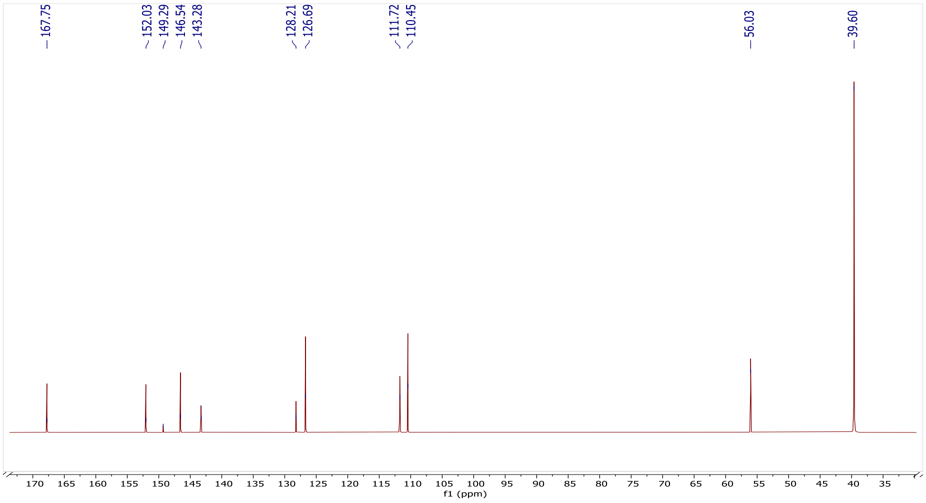

6a

FT-IR

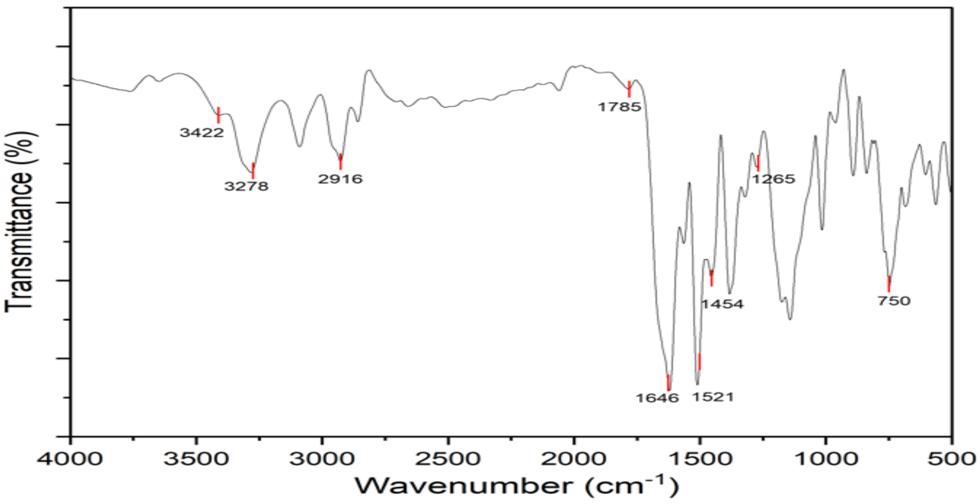

^{1}HNMR

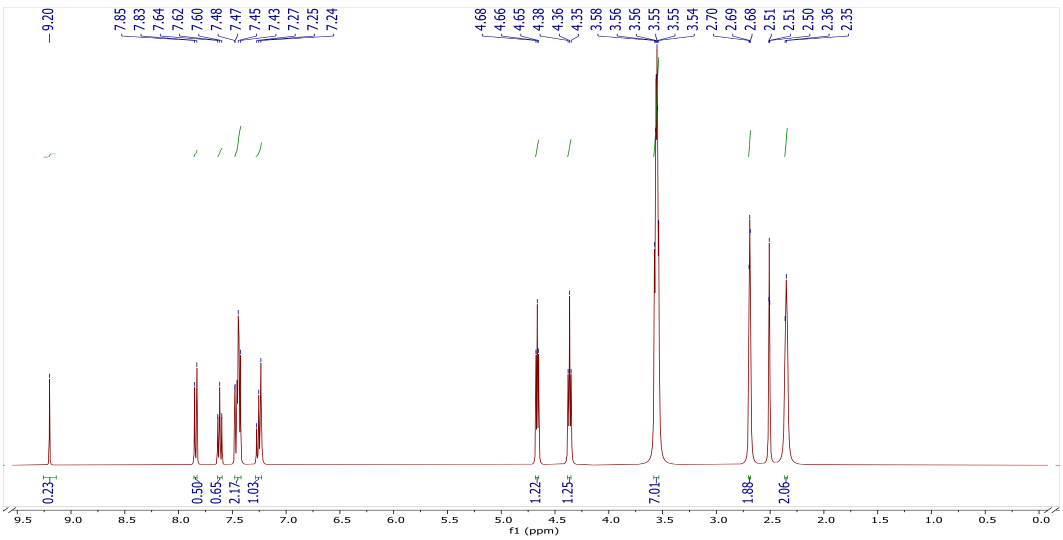

^{13} CNMR

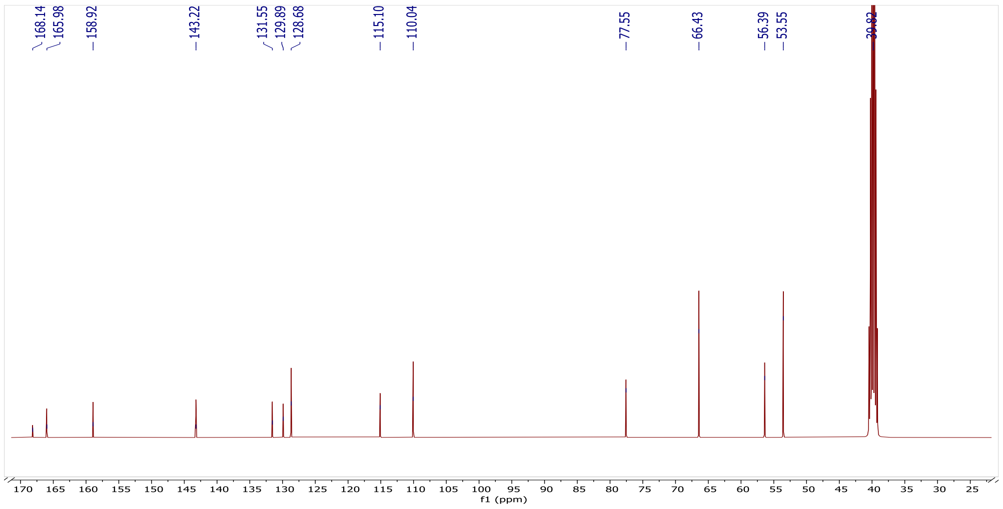

6b

FT-IR

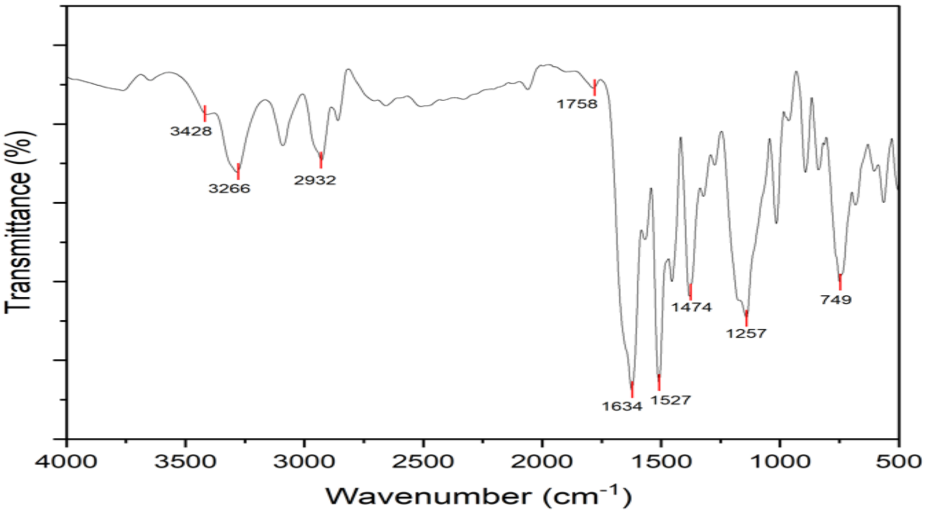

^{1}HNMR

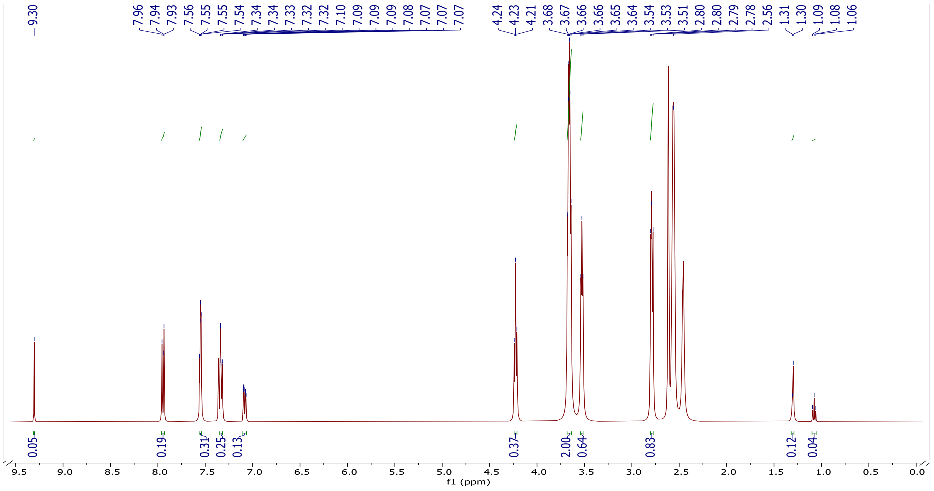

^{13} CNMR

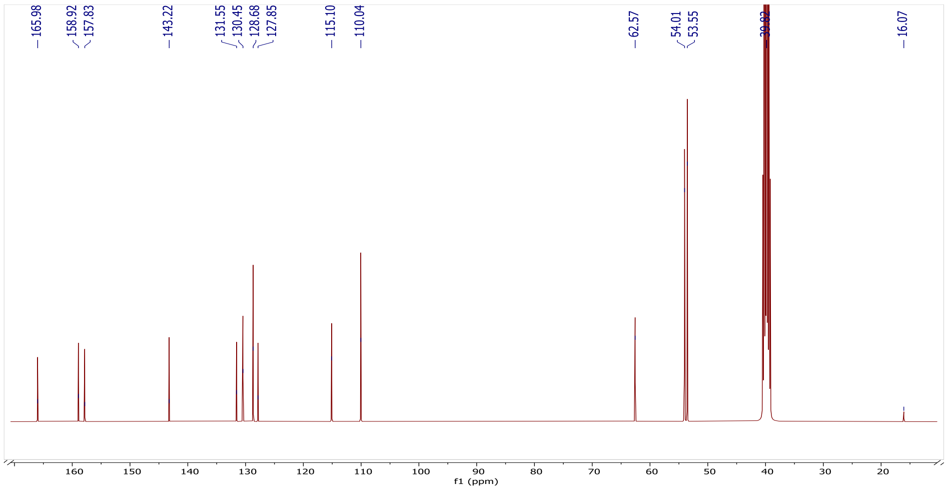

6c

FT-IR

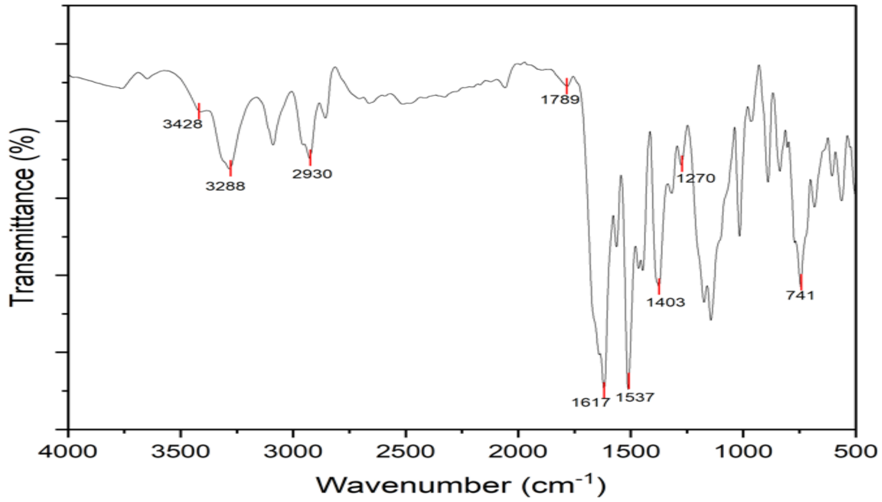

^{1}HNMR

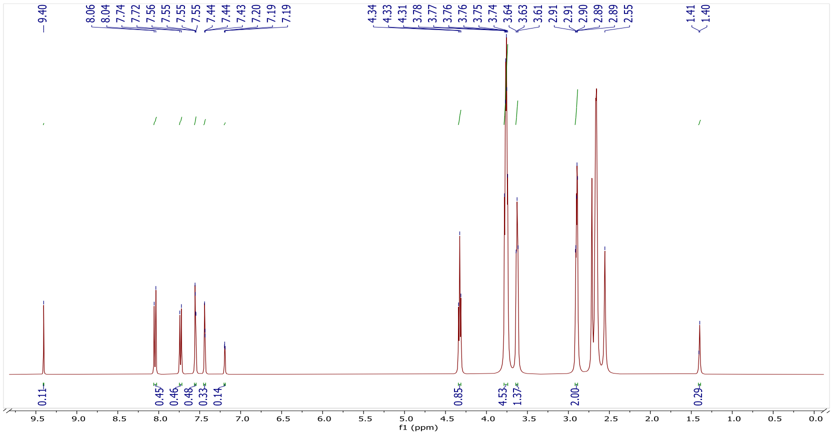

[13] CNMR

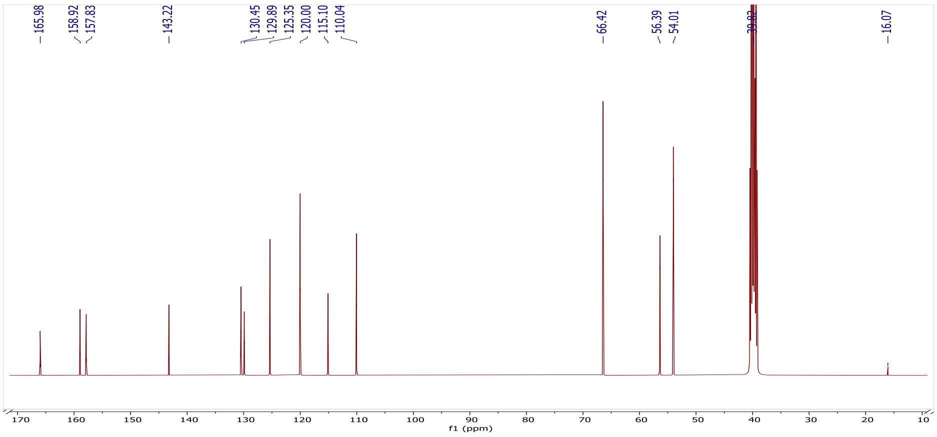

6d

FT-IR

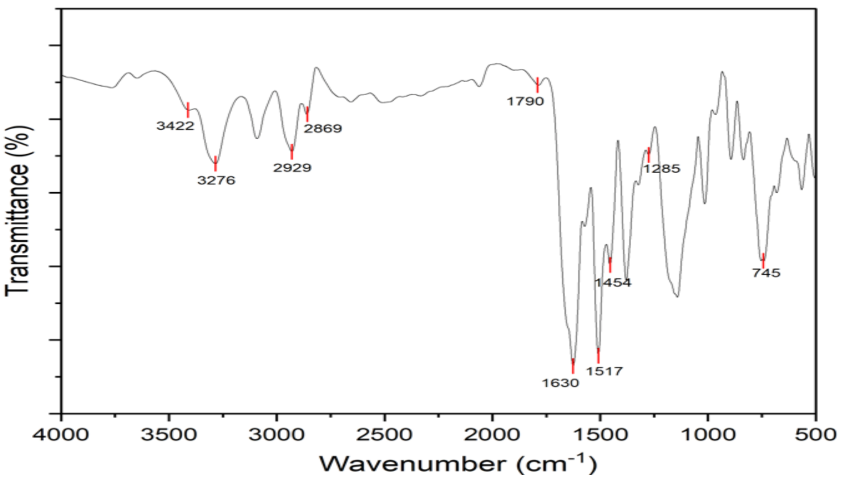

^{1}HNMR

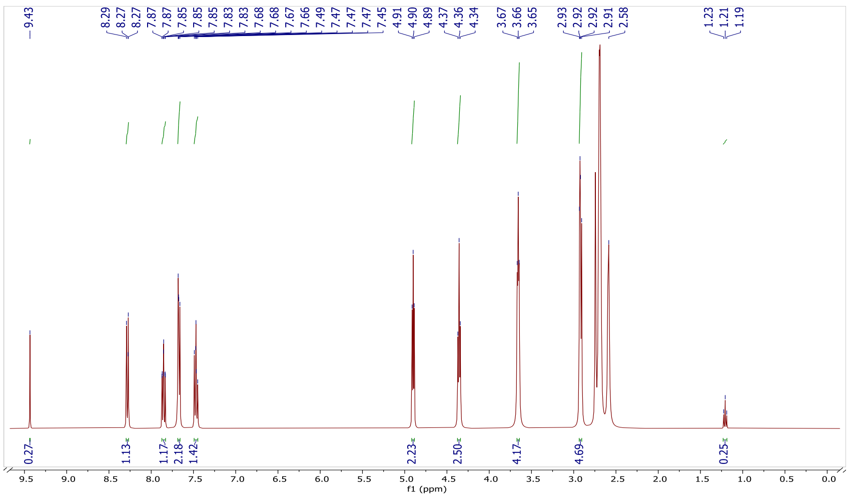

[13] CNMR

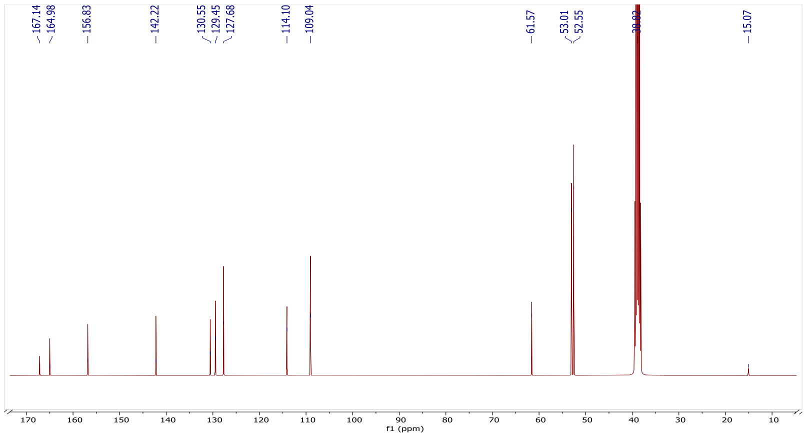

6e

FT-IR

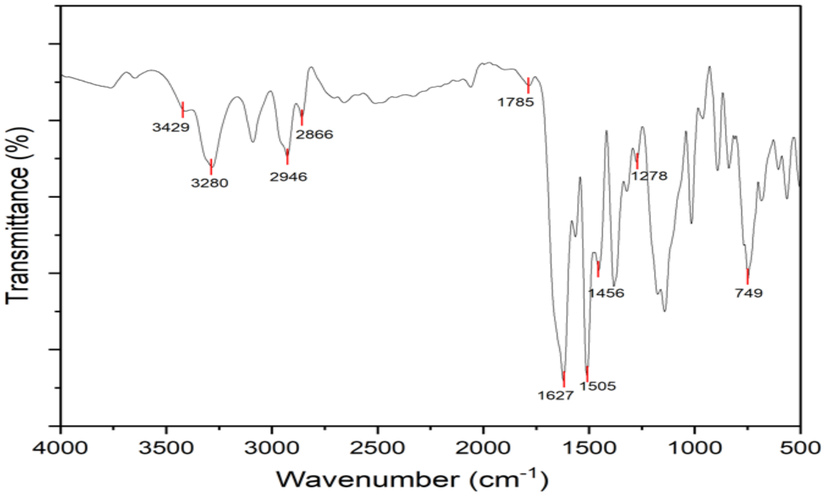

^{1}HNMR

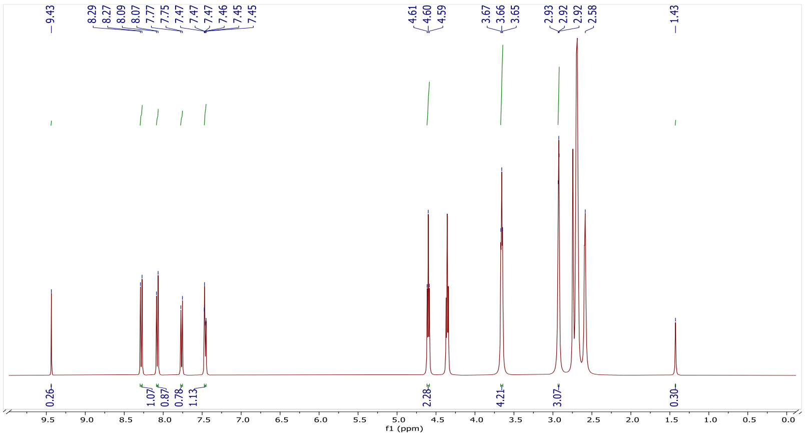

^{13} CNMR

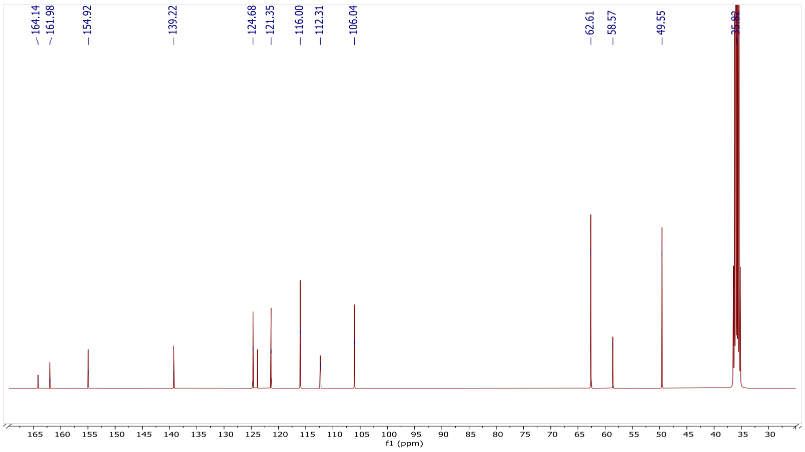

7a

FT-IR

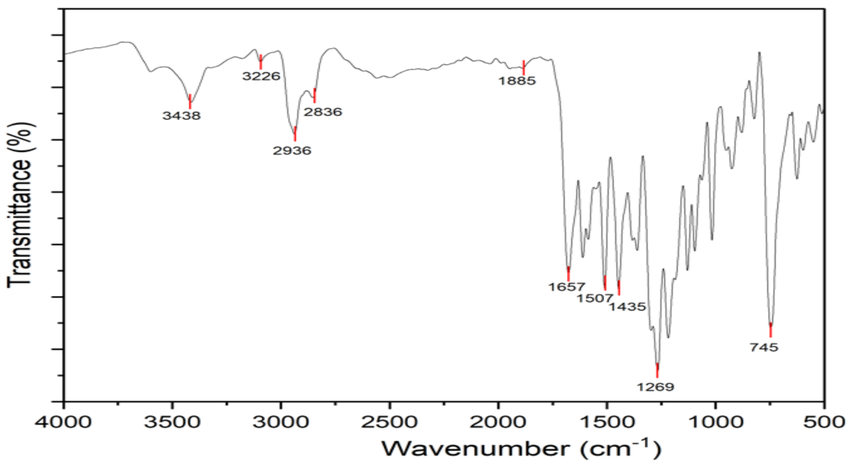

^{1}HNMR

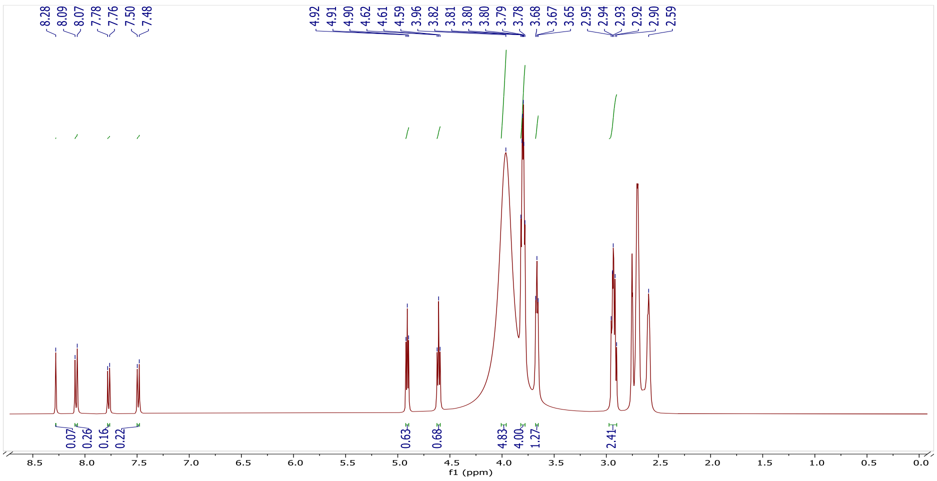

[13] CNMR

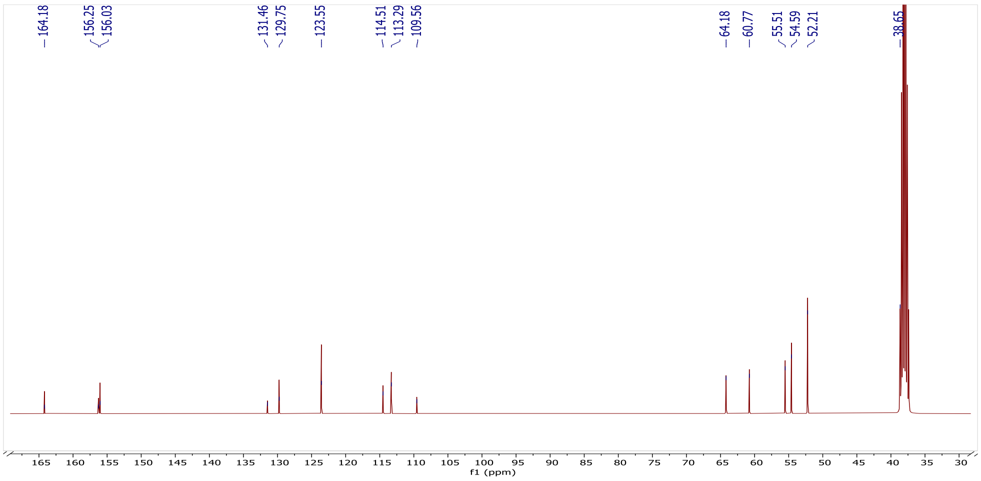

7b

FT-IR

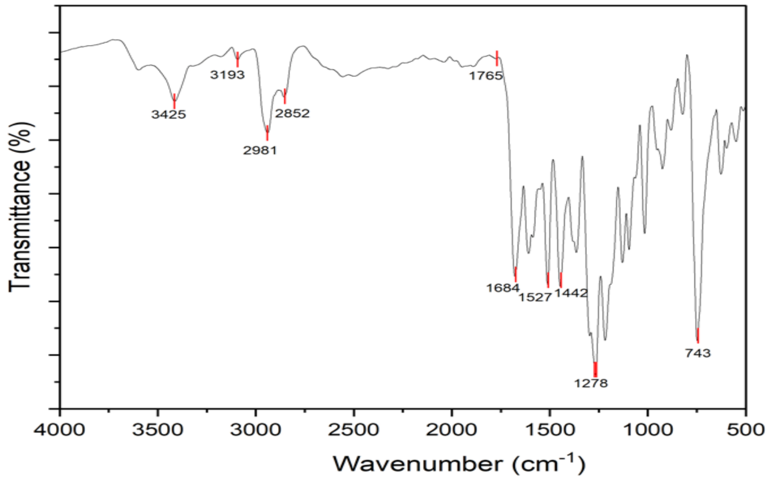

^{1}HNMR

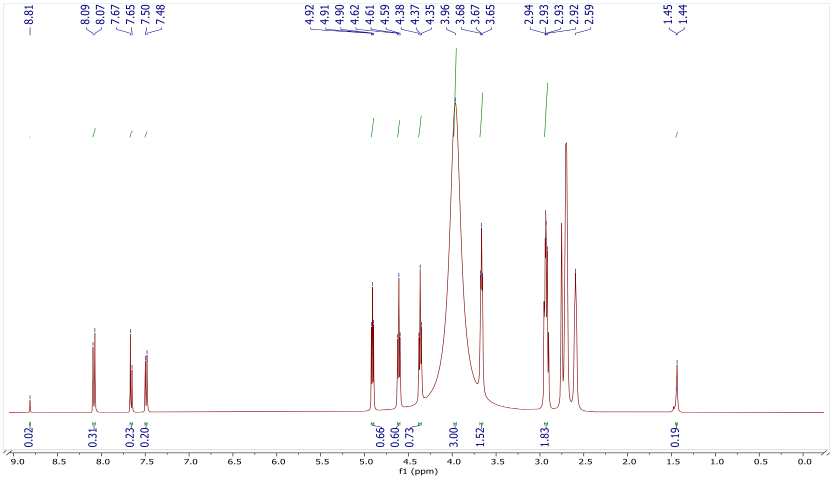

[13] CNMR

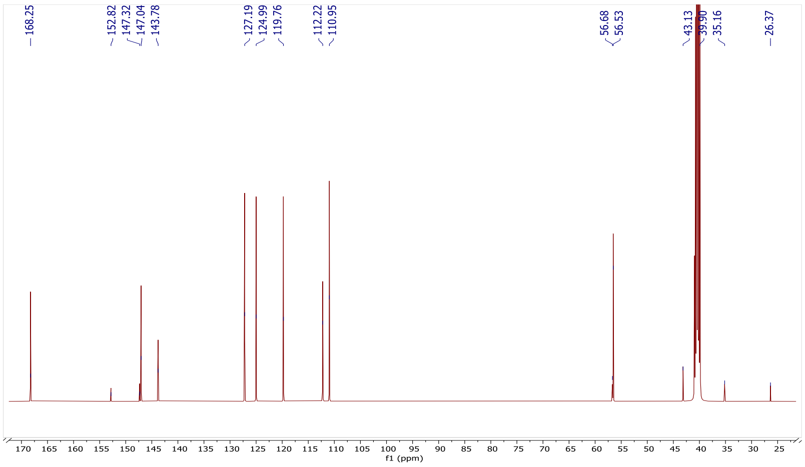

7c

FT-IR

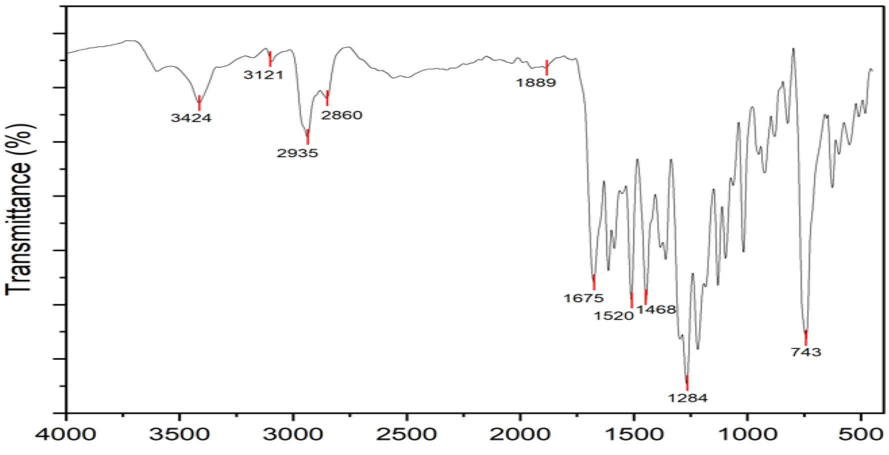

^{1}HNMR

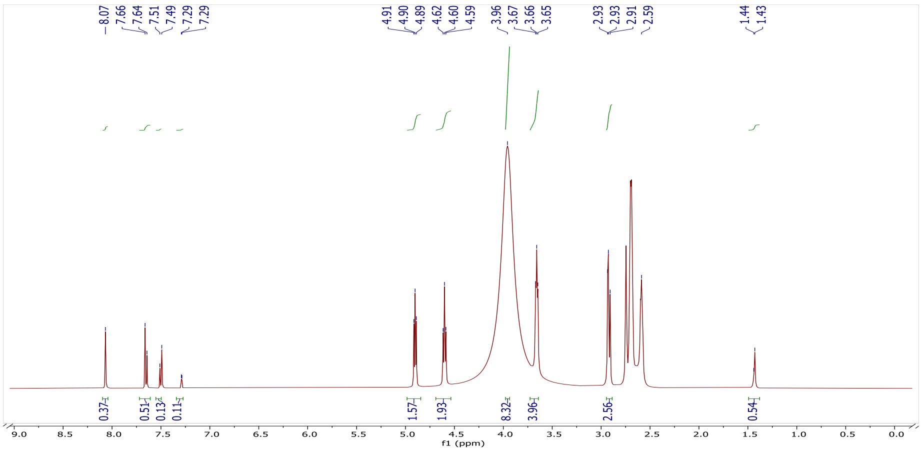

^{13} CNMR

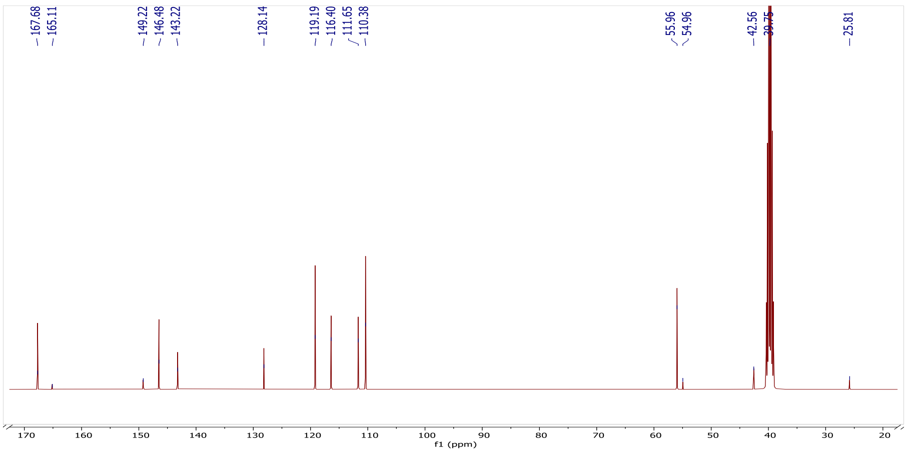

7d

FT-IR

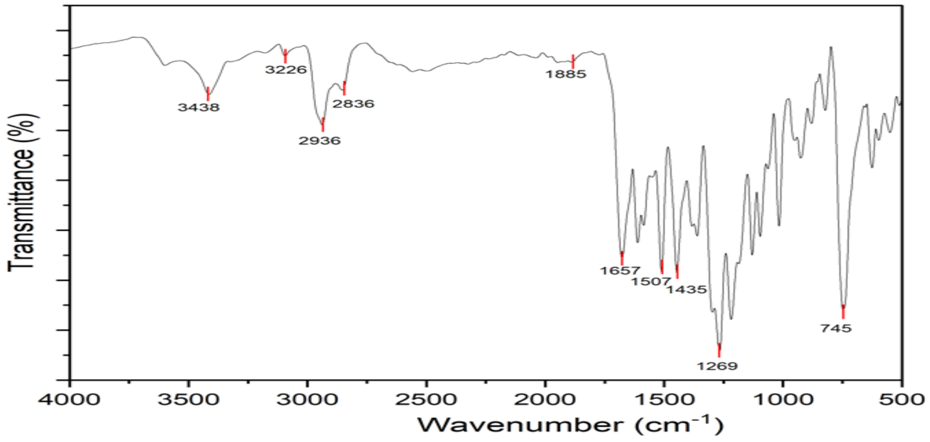

^{1}HNMR

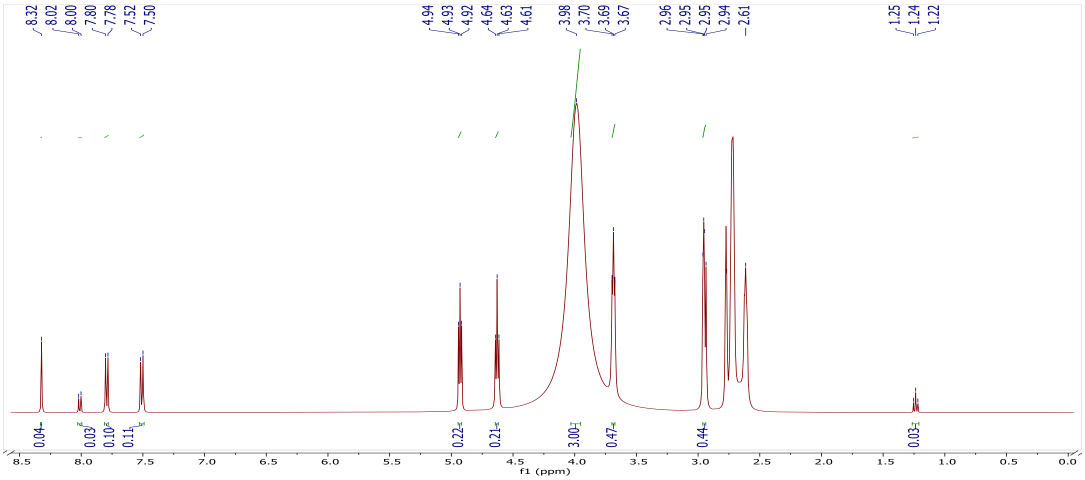

^{13} CNMR

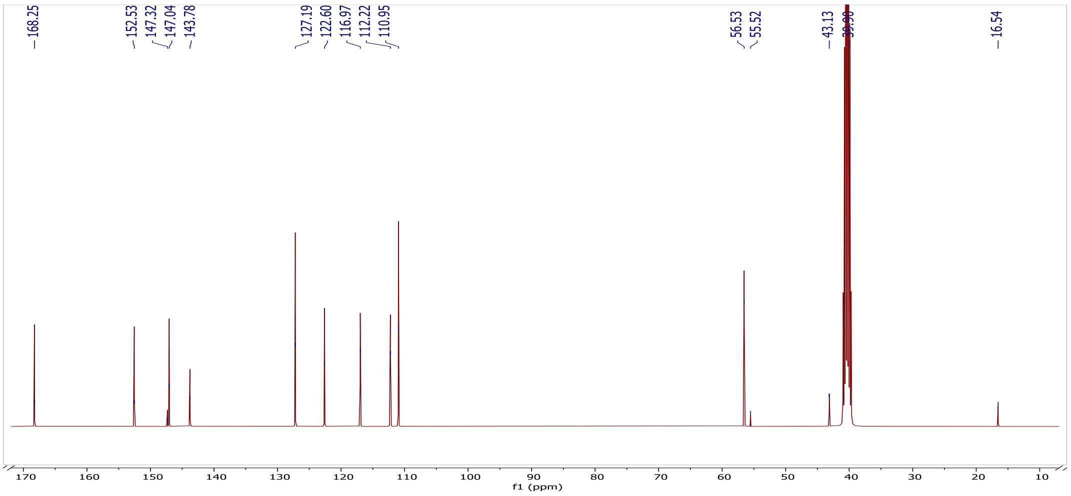

7e

FT-IR

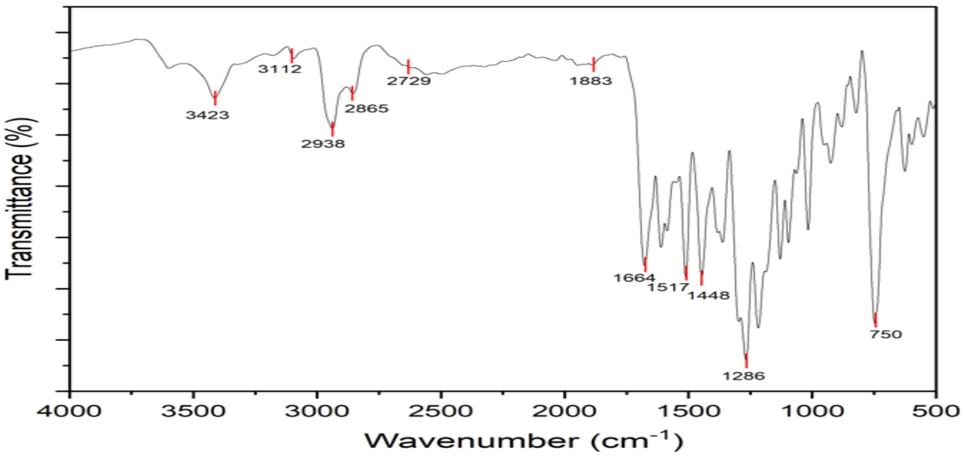

^{1}HNMR

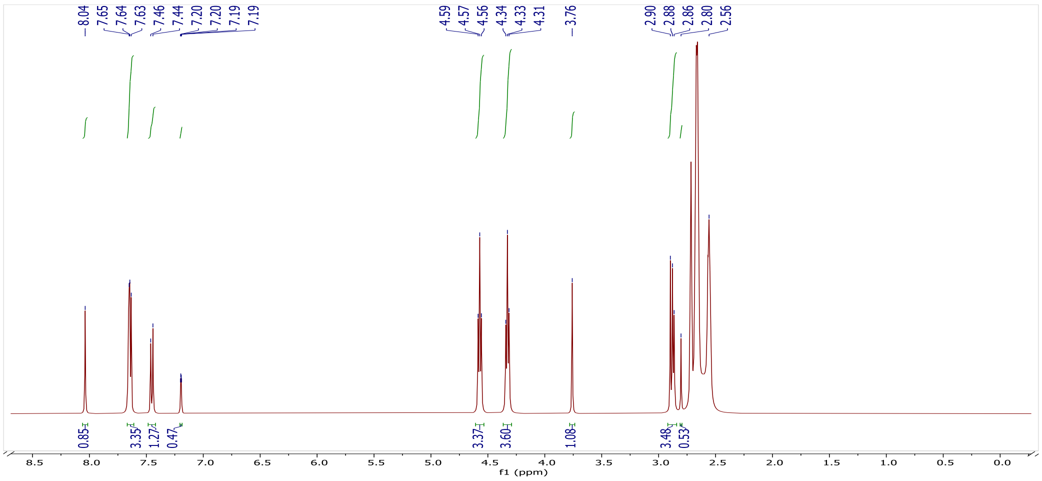

^{13} CNMR

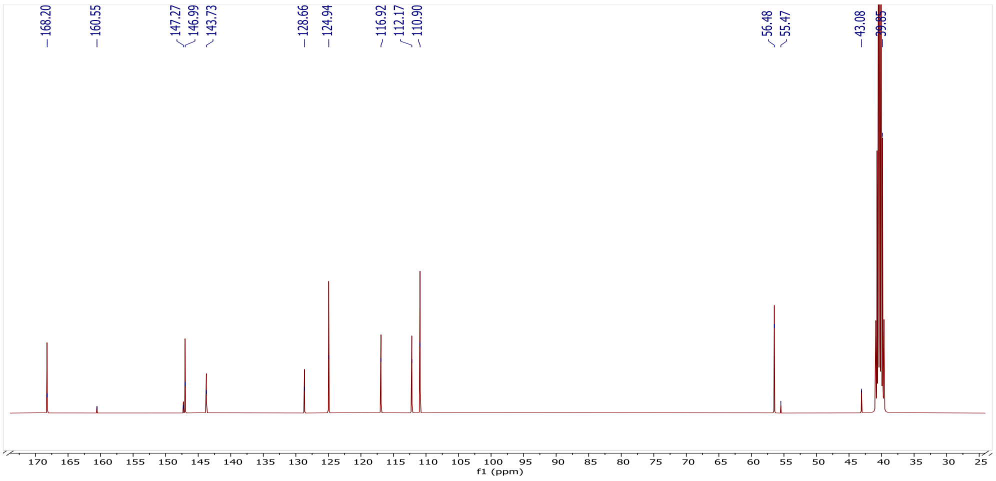

Supplement: Supplementary file 2 [file DataSheet3.pdf]
