# Supplementary material for: Design, synthesis, and in vitro, in vivo, and in silico evaluation of novel substituted 1,3,4-thiadiazole derivatives as anticonvulsant agents
Source: Front Chem. 2025 Feb 12;12:1515866. doi: 10.3389/fchem.2024.1515866 (PMC11861159; doi:10.3389/fchem.2024.1515866)
Supplement: Supplementary file 3 [file DataSheet1.pdf]

Design, synthesis, in-vitro, in-vivo, and in-silico evaluation of novel substituted 1,3,4-thiadiazole derivatives as anticonvulsant agents

Tulika Anthwal[1], Swati Pant[1], Preeti Rana[2], Sumitra Nain[1]*

1. Department of Pharmacy, Banasthali Vidyapith, Banasthali, Rajasthan, India, 304022
2. National Institute of Pharmaceutical Education and Research (NIPER), Balanagar, Hyderabad, Telangana, India

***Correspondance**: Dr. Sumitra Nain, Department of Pharmacy, Banasthali Vidyapith, Banasthli, Rajasthan, India, 304022.
Email: nainsumitra@gmail.com

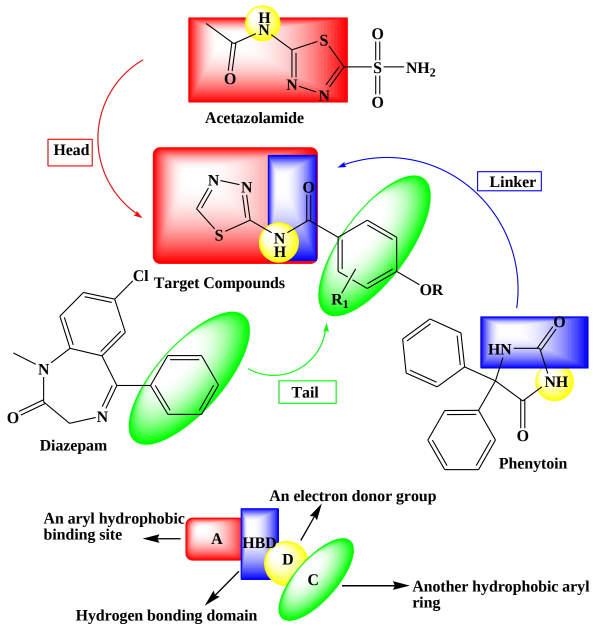

Figure 1. Strategy for designing titled compounds and their pharmacophore patterns

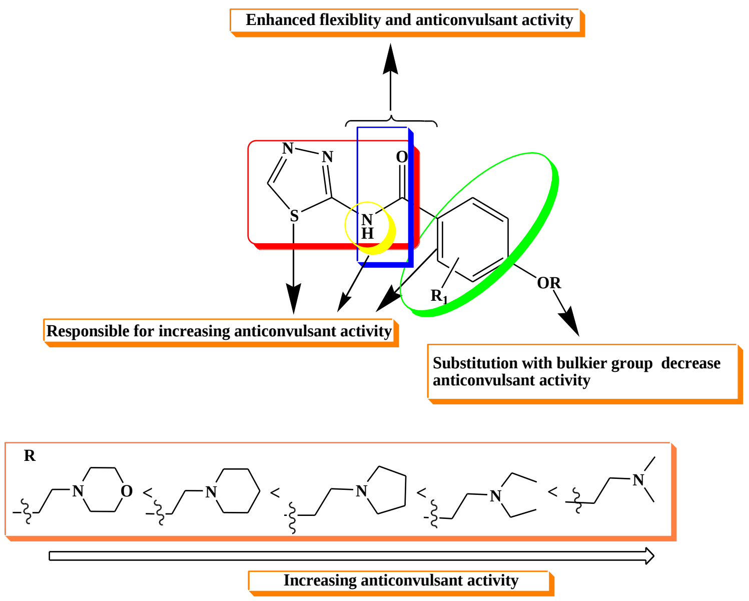

Figure 2. SAR of synthesized compounds

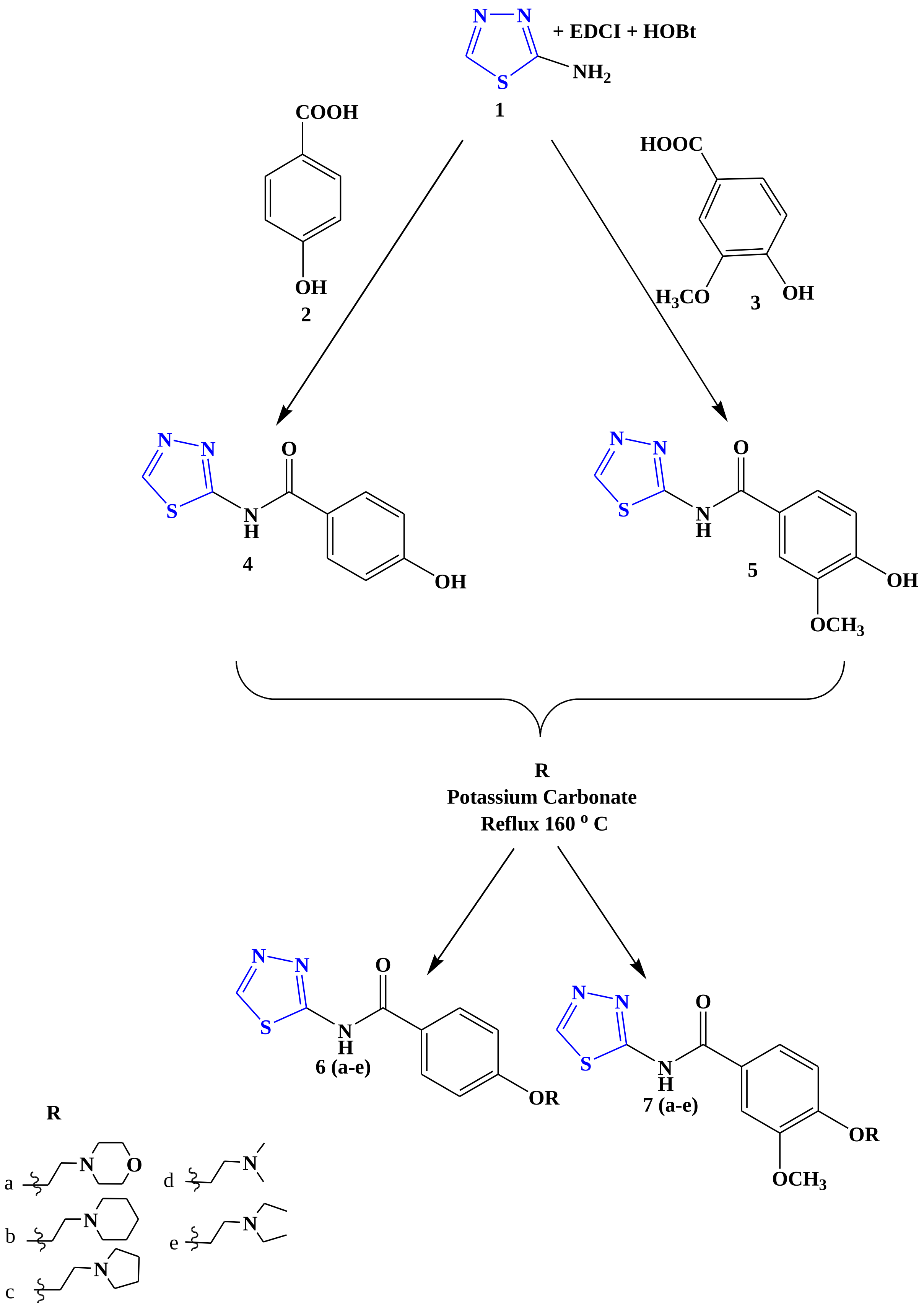

Scheme 1 Systematic scheme for synthesizing the target compounds **6(a-e)** and **7(a-e)**

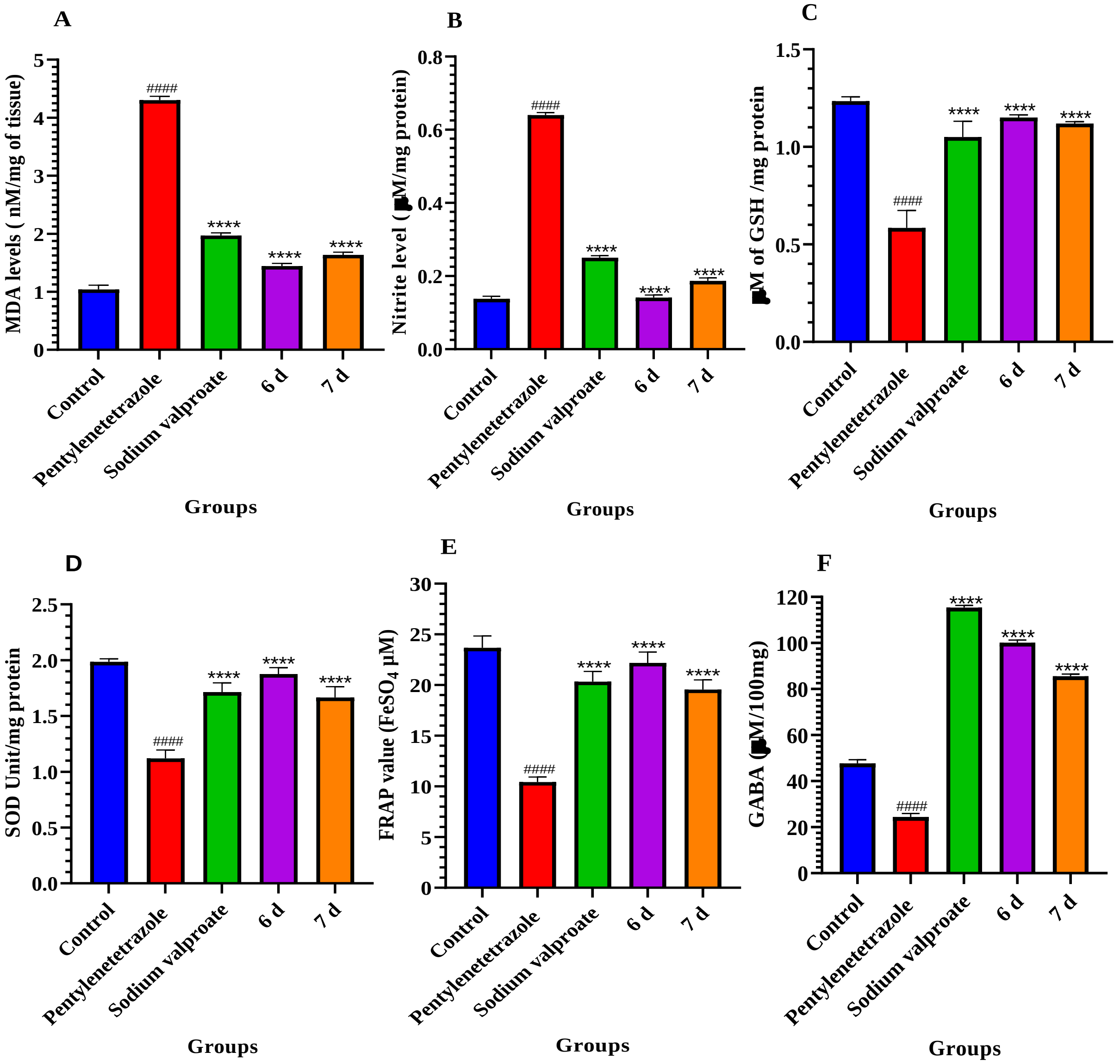

Figure 3. Effect of compounds 6d and 7d lipid peroxidation (A), nitrite level (B), glutathione content (C), superoxide dismutase (D), total antioxidant capacity (E) and GABA level (F) compared to the pentylenetetrazole treated group of mice. Values are expressed as Mean±SD, n=6. ####p < 0.0001 (positive control is compared with the normal control), ****p < 0.0001 (treatment groups are compared with positive control).

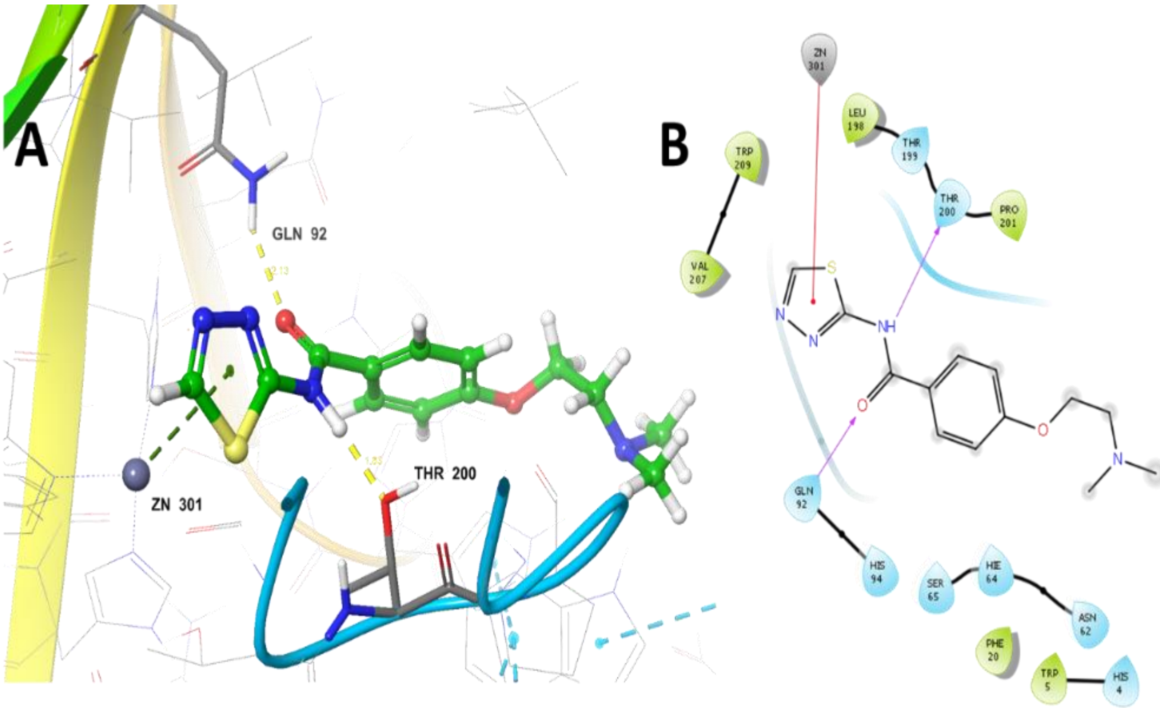

Figure 4. Represents docking studies of compound **6d** into active site human carbonic anhydrase IX (PDB-ID-5SZ5) (**A**) 3D docking model (**B**) 2D docking model

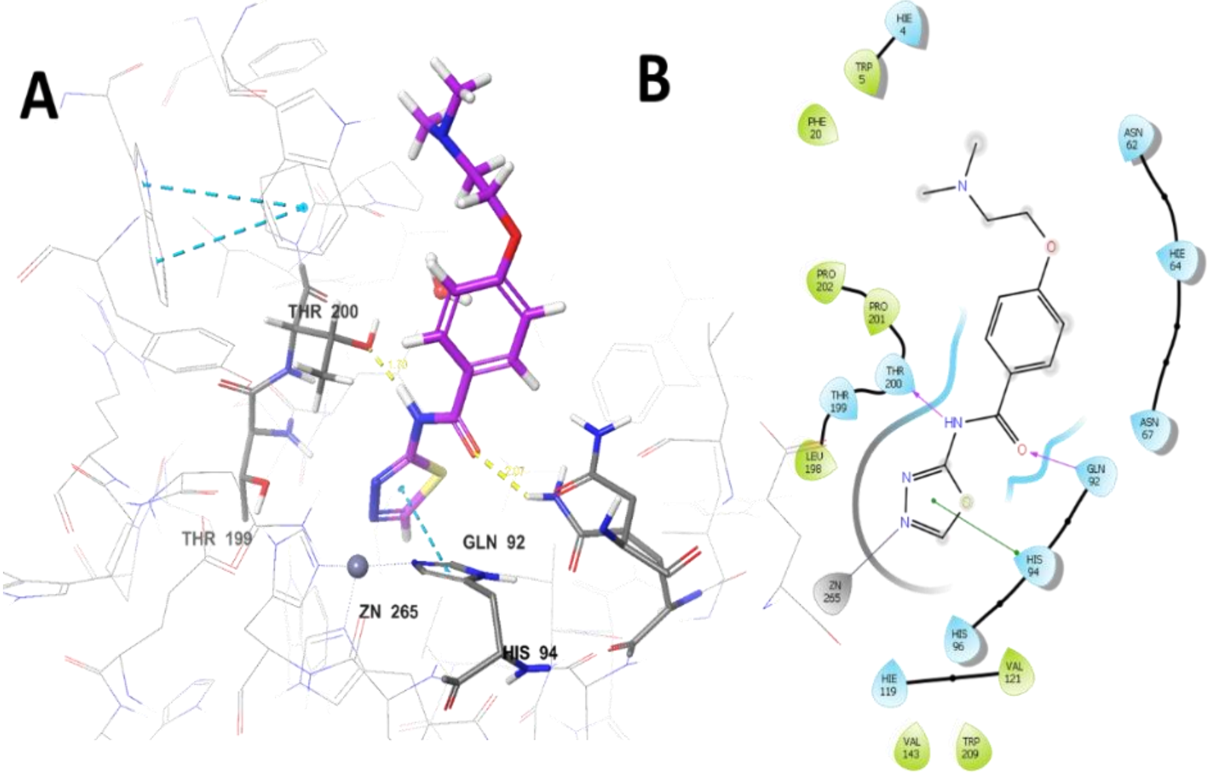

Figure 5. Represents docking studies of compound **6d** into active site human carbonic anhydrase II (PDB-ID-5AML) (**A**) 3D docking model (**B**) 2D docking mode
